# The Essentiality of Arachidonic Acid in Infant Development

**DOI:** 10.3390/nu8040216

**Published:** 2016-04-12

**Authors:** Kevin B. Hadley, Alan S. Ryan, Stewart Forsyth, Sheila Gautier, Norman Salem

**Affiliations:** 1 DSM Nutritional Products, 6480 Dobbin Road, Columbia, MD 21045, USA; kevin.hadley@dsm.com (K.B.H.); sheila.gautier@dsm.com (S.G.); norman.salem@dsm.com (N.S.Jr.); 2 Clinical Research Consulting, 9809 Halston Manor, Boynton Beach, FL 33473, USA; 3 School of Medicine, Dentistry & Nursing, University of Dundee, Ninewells Hospital and Medical School, Dundee, UK; stewartforsyth@btinternet.com

**Keywords:** arachidonic acid, docosahexaenoic acid, infant formula, growth, human milk, long-chain polyunsaturated fatty acids

## Abstract

Arachidonic acid (ARA, 20:4*n*-6) is an *n*-6 polyunsaturated 20-carbon fatty acid formed by the biosynthesis from linoleic acid (LA, 18:2*n*-6). This review considers the essential role that ARA plays in infant development. ARA is always present in human milk at a relatively fixed level and is accumulated in tissues throughout the body where it serves several important functions. Without the provision of preformed ARA in human milk or infant formula the growing infant cannot maintain ARA levels from synthetic pathways alone that are sufficient to meet metabolic demand. During late infancy and early childhood the amount of dietary ARA provided by solid foods is low. ARA serves as a precursor to leukotrienes, prostaglandins, and thromboxanes, collectively known as eicosanoids which are important for immunity and immune response. There is strong evidence based on animal and human studies that ARA is critical for infant growth, brain development, and health. These studies also demonstrate the importance of balancing the amounts of ARA and DHA as too much DHA may suppress the benefits provided by ARA. Both ARA and DHA have been added to infant formulas and follow-on formulas for more than two decades. The amounts and ratios of ARA and DHA needed in infant formula are discussed based on an in depth review of the available scientific evidence.

## 1. Introduction

During the first year of life, infants have special nutritional requirements to maintain a healthy body and support rapid growth and development. Human milk is typically the sole source of nutrition that must supply the infant with appropriate amounts of energy and nutrients. The long-chain polyunsaturated fatty acids (LCPUFA), docosahexaenoic acid (DHA, 22:6*n*-3) and arachidonic acid (ARA, 20:4*n*-6) are always present in human milk. These fatty acids play key roles in the structure and function of human tissues, immune function, and brain and retinal development during gestation and infancy [1,2]. Although breastfeeding is considered the ideal way to nourish infants, recent nutrition surveys report that the majority of infants in developed countries receive at least some infant formula during the first year of life [3,4].

Both ARA and DHA have been added to infant formulas in the United States since 2001, although supplementation began in Europe much earlier. Most infant formulas contain 0.2% to 0.4% of total fatty acids as DHA and between 0.35% and 0.7% of total fatty acids as ARA based on worldwide averages of DHA and ARA content in human milk [5] and the recommendations from a number of international expert groups [6,7,8,9]. Thus, all commercially available infant formulas contain preformed ARA at levels equal to or higher than the DHA content in order to maintain adequate DHA and ARA status in non-breastfed infants.

Both α-linolenic acid (ALA, 18:3*n*-3) and linoleic acid (LA, 18:2*n*-6) are regarded as nutritionally essential fatty acids [10]. However, as Lauritzen *et al.* [10] point out, all classic signs of essential fatty acid (EFA) deficiency can be completely reversed by the administration of *n*-6 fatty acids alone, particularly ARA. With respect to infants, the presence of a relatively fixed level of preformed ARA in human milk and the active accumulation of ARA by tissues throughout the body support the concept of the essentiality of ARA. Previously, a description of the essentiality of ARA during infancy has not been considered in detail, although a brief outline of the essentiality of *n*-6 and *n*-3 polyunsaturated fatty acids was presented by Lauritzen *et al.* [10] in 2001.

The purpose of this paper is to review the essentiality of ARA for infant growth and development. We consider both animal models and human studies of ARA. We describe: (1) ARA accumulation and function in brain and tissues; (2) ARA content in human milk and in various tissues, including rates of accretion during gestation and early infancy; (3) the structure and biosynthesis of ARA from LA and its role as a precursor to leukotrienes, prostaglandins, and thromboxanes, collectively known as eicosanoids; (4) dietary intakes of ARA during late infancy and early childhood when non-breast milk food items are introduced into the diet; (5) immune system development and the dual role of PGE_2_ and its receptors in modulating the inflammatory response during infancy; (6) bone metabolism and growth; (7) regulation of cardiac function; (8) consequences of ARA deficiency; (9) the importance of ARA for optimal brain and central nervous system development; (10) the history, reasons for, and nutritional effects of adding both DHA and ARA to infant formulas, with an emphasis on the effects of ARA; (11) the importance of ARA in infant health; and (12) the regulatory requirements for ARA and DHA in infant formulas. Based on a detailed review of the scientific literature presented herein, recommendations for dietary intakes ARA during infancy are provided.

## 2. ARA Accumulation and Function in Brain and Tissue

Over the last decade, there has been increased understanding of the molecular roles that the *n*-3 and *n*-6 PUFA play in brain and cellular function. The variety of functions shown to be related to ARA indicates its importance and essentiality in the metabolic chain of events leading to brain structural lipid development, signaling, and many basic cellular functions.

ARA is indispensable for brain growth where it plays an important role in cell division and signaling [11]. The brain in mammals consists of 60% fat, which requires DHA and ARA for its growth and function [12]. Across different species of mammals there is little variation in DHA and ARA composition of the brain. ARA is one of the most abundant fatty acids in the brain, and compared with DHA, ARA is present in similar quantities [13,14]. The two fatty acids account for approximately ~25% of its total fatty acid content predominately in the form of phospholipids and thus are major structural components of neural cellular membranes.

**Figure 1:**
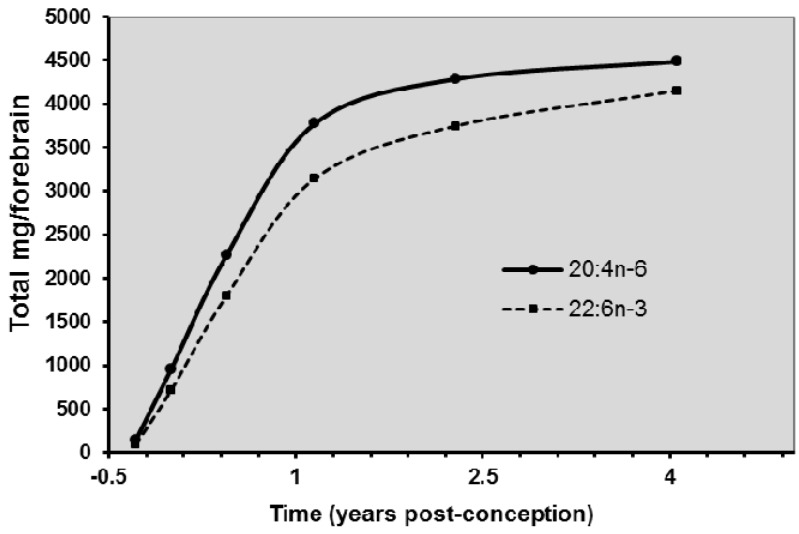
Long-chain polyunsaturated fatty acids (LCPUFA) accretion in the human brain during perinatal development (Data from Martinez [15]).

ARA rapidly accumulates in the brain during development [1,14,15] which takes place from the beginning of the third trimester of gestation up to about 2 years of age [16] (Figure 1). As shown in brain kinetics in fetal baboons, [17] in addition to maternal preformed ARA, LA may be transported across the blood-brain barrier despite its very low content within brain lipids. The brain has an active desaturation/elongation system that converts LA to ARA [17]. ARA activity is higher in brain than in other organs such as the liver. However, the conversion of LA to ARA is low (see below).

The maximum rate of brain growth is primarily associated with myelination [14]. In animal models, approximately 50% of the adult amounts of ARA and DHA accumulate in rat brain during the period before myelination and at 15 days after birth when myelination has just started [14]. Diets low in LCPUFA adversely affect the development of the myelin lipids needed early in brain development [14].

ARA has several functions in the brain. ARA mediates neuronal firing [18], signaling [19], and long-term potentiation [20]. ARA also helps maintain membrane order and hippocampal plasticity [21], defends the brain against oxidative stress in the hippocampus by activating the peroxisome proliferator-activated receptor gamma (PPARγ), and aids in the synthesis of new protein in tissue [22].

A potentially important aspect of ARA metabolism *in vivo* is its function as an immediate precursor for adrenic acid (22:4*n*-6) [23]. Adrenic acid is the third most abundant PUFA in the brain that is found in large quantities in myelin lipids, particularly in phosphatidylethanolamine (PE) [1]. Rapid accumulation of adrenic acid, like ARA, occurs during the early post-natal period of the brain growth spurt in infants. The conversion of ARA to adrenic acid may represent an important pathway for ARA utilization in infants in order to meet the rapid increase of adrenic acid needed for neural tissue development.

Using a single dose of U-^13^C-labeled ARA to investigate preformed ARA utilization in baboon neonates, Wijendran *et al.* [23] reported that a major portion of ARA consumed (79%–93%) was accumulated as ARA in tissue lipids, consistent with its primary function as a principal constituent of membrane lipids. Approximately 5% to 16% of ARA was converted to adrenic acid. Based on tracer data, net accretion of ARA and adrenic acid during the first 4 weeks of age in the neonate baboon brain was 17% and 8%, respectively, corresponding to efficiencies (*i.e.*, percentage of dose recovered in brain) of 0.48% and 0.54% of dietary levels, respectively.

To determine the effects that differing DHA to ARA ratios have on tissue fatty acids, twelve-week-old full term baboons were randomized to one of three diets: control (no DHA or ARA), moderate (0.33% DHA, 0.67% ARA) and high LCPUFA (1.00% DHA, 0.67% ARA) [24]. In all groups, DHA levels increased significantly in liver, heart, plasma and in the central nervous system (CNS) regions (precentral gyrus, frontal cortex, inferior and superior colliculi, globus pallidus, and caudate). The formula with the highest level of DHA significantly reduced ARA levels in two areas of the brain (superior colliculus and globus pallidus), indicating its competition with ARA and the importance of a proper balance of DHA to ARA.

Phosphatidylcholine (PC) is a lipid class that is a major component of most intracellular membranes [25]. Some intracellular lipid bilayers include PC containing ARA (ARA-PC). ARA-PC functions as a retrograde messenger in long-term potentiation of synapses in the hippocampus CA1 region [26] and is involved in migration of neurons in the cerebral cortex [27].

Using imaging mass spectrometry, Yang *et al.* [25] characterized the distribution of ARA-PC within cultured neurons of the superior cervical ganglia and found an increasing gradient of ARA-PC along the proximodistal axonal axis that may provide a source for free ARA release [25]. Released free ARA is known to activate protein kinases and ion channels, inhibit neurotransmitter uptake, and enhance synaptic transmission [11]. Free ARA therefore modulates neuronal excitability. As ARA mediates intracellular signaling the concentration of free ARA must be maintained at precise levels within the cells. A higher concentration of ARA-PC near the axon terminal might provide a timely source of ARA when needed during the activated period [25].

ARA also is responsible for the activation of syntaxin-3 (STX-3), a plasma membrane protein involved in the growth and repair of neurites [28]. Growth of neurite processes from the cell body is a critical step in neuronal development. STX-3 serves as a single effector molecule and direct target for ARA [28]. Neurite growth closely correlates with the ability of ARA to activate STX-3 in membrane expansion at growth cones [28].

ARA also enhances the engagement of STX-3 with the fusogenic soluble N-ethylmaleimide-sensitive factor attachment protein receptors (SNARE complex), proteins that form a ternary complex that drives exocytosis [29]. In the brain, at the neuromuscular junction, and in endocrine organs, a set of three SNARE proteins has a primary role in producing fusion of vesicular and plasma membranes. The formation of this SNARE complex drives membrane fusion which leads to the release of vesicular cargo into the extracellular spaces [29]. Darios and colleagues [29] report that α-synuclein, a synaptic modulatory protein implicated in the development of Parkinson disease, can sequester ARA and thereby block the activation of the SNARE complex. This finding underlines the importance of ARA for the regulation of synaptic transmission and transport.

Detergent resistant microdomains, also referred to as lipid rafts, are specialized regions within plasma membranes [30]. These microdomains serve as platforms for biomechanical interactions between the lipid and protein components of signal transduction pathways [30,31,32]. The outer leaflet of lipid rafts is highly enriched with glycol-sphingolipids and cholesterol [32]. The inner, or cytosol facing leaflet is enriched with alkenyl forms of PE which have been termed plasmenylethanolamine. Electrospray ionization/mass spectrometric analysis has shown that the ARA-containing plasmenylethanolamine represents as much as 50% of the phospholipids of the cytosolic leaflet [31]. This is consistent with a role of PE as an important source of ARA within the cell.

Stearoyl-2-arachidonoyl is a highly abundant species of phosphatidylinositol (PI) found in the phosphorylated forms of PI, the phosphoinositides [33,34,35,36,37,38]. In addition to serving as a substrate for phospholipase C to produce inositol-triphosphate and diacylglycerol, phosphoinositides serve important biochemical functions including lipid signaling, cell signaling and membrane trafficking. Phosphoinositides perform these roles in part by serving as adaptors for protein-protein and protein-membrane interactions in order to facilitate and/or regulate G-receptor protein activity and signal transduction, and trafficking of various metabolites such as cholesterol or calcium, or other ions, between cellular compartments [39,40,41,42,43]. These biochemical functions of ARA demonstrate its importance for cell signaling, trafficking and regulation of spatial-temporal interactions between cellular structures.

## 3. Levels of ARA in Human Milk, Brain, and Tissues

Fat is a critical component of human milk that provides energy and nutrients needed for the development of the CNS [10]. DHA and ARA are the principal LCPUFA found in human milk. The synthesis of DHA and ARA is limited in infants [5] and both DHA and ARA must be obtained from dietary sources. Amounts of DHA and ARA in human milk tend to vary by diet, nutritional status, and other factors [5]. Based on data from 65 studies of human milk from 2474 women, the mean concentration of ARA (by weight) was 0.47% ± 0.13% (range 0.24% to 1.0%) whereas the mean concentration of DHA was 0.32% ± 0.22% (range 0.06% to 1.4%) [5]. The DHA concentration in human milk is lower and more variable than ARA. The level of ARA in human milk is much more stable. The relatively stable content of ARA in human milk is biologically important because it provides preformed ARA consistently at a time when brain growth and development is most critical. The majority of ARA in human milk does not derive from dietary LA but rather from maternal stores of ARA [44]. The correlation between DHA and ARA is low, which may reflect a higher degree of variability in the ratio of DHA to ARA in individual human milk samples [5].

The composition of the brain is dominated by ARA and DHA [45]. During pregnancy, both ARA and DHA are preferentially transferred across the placenta [46] and sequestered in the developing brain from the earliest phases of its growth. After birth, human milk provides both DHA and ARA to the breastfed infant [47] with a rapid rise towards adult levels of DHA and ARA in the brain within the first two years of life [48]. ARA is found at a level comparable to that of DHA in neural membranes, particularly those of the brain [49].

Based on estimated total body content of ARA from fetal organ weights during the last trimester of pregnancy and early infancy the relative amount of brain ARA decreases, but because of brain growth the absolute amount of ARA increases [50]. In fact, the absolute amount of ARA increases in all organs with increasing gestational age while the relative contribution (g per 100 g fatty acids; g %) decreases [50]. At 25 weeks gestation, the whole fetal body contains about 1.1 g ARA which increases to 4.2 g ARA at 35 weeks gestation. A full-term infant (3500 g) has about 7.6 g of ARA. The accretion rate of ARA is estimated to be 6.1 mg/day during the first 25 weeks and increases to 95.2 mg/day by 35–40 weeks gestation. The fetal accretion rate for ARA is 2-fold that of DHA [50]. Most of the bodily ARA at 25 and 40 weeks is located in skeletal muscle, adipose tissue and the brain, in that order [50].

In human infant central nervous tissue (cerebral cortex and retina) ARA comprises approximately 10%–12% of total fatty acids [49]. The amount of ARA in central nervous tissue appears to be influenced to a greater extent by postnatal age than by dietary ARA supply [49]. Samples of frontal cerebral cortex obtained from 58 human autopsies (mean age 40 ± 29 years) indicated that the relative levels of PUFA expressed as a percentage of total fatty acids generally decrease with age with the exception of DHA [51].

**Figure 2:**
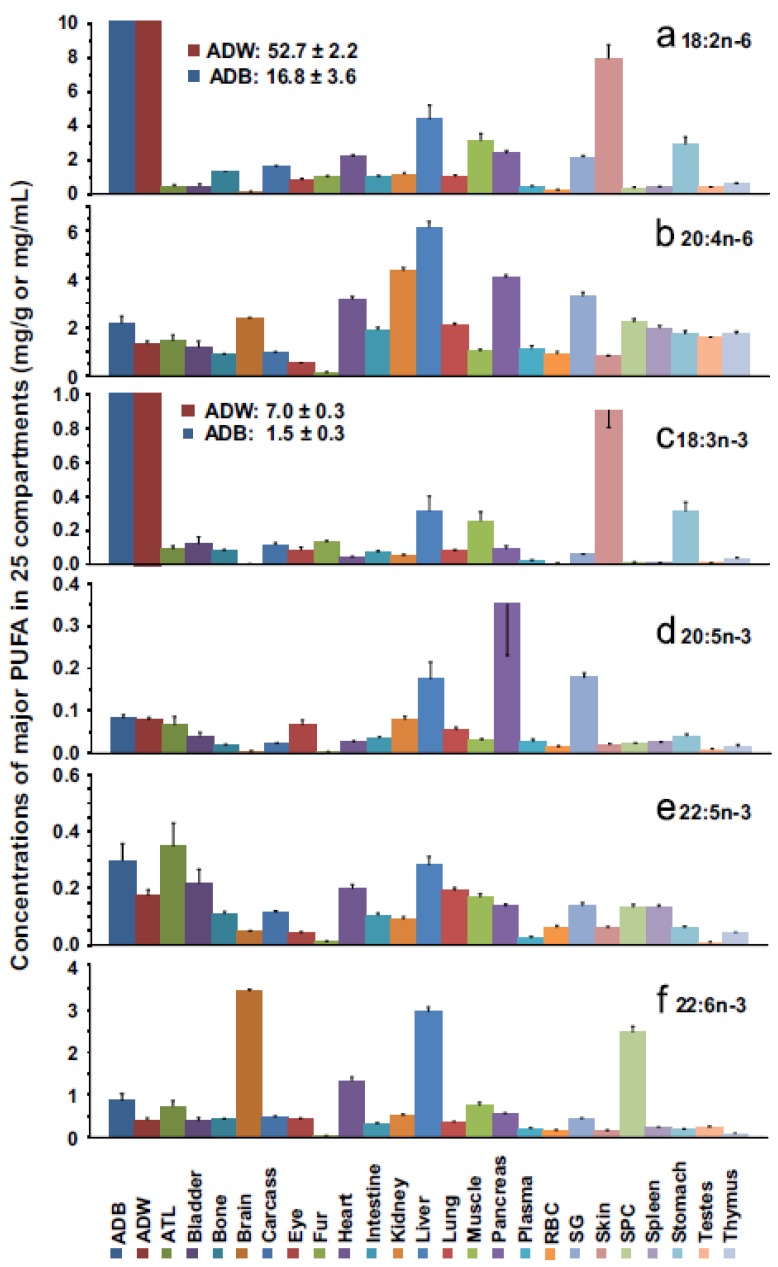
Distribution of fatty acids in 25 different tissue compartments in young male rats. Abbreviations: ATL, adrenal gland, thyroid gland, mandibular gland, and lymph nodes; RBC, red blood cell; SG, salivary gland; ADB, brown adipose tissue; ADW, white adipose tissue (from Salem *et al.* [52]).

The distribution of *n*-6 and *n*-3 PUFA was determined in various viscera and tissues within the whole body of rats fed a diet containing 10 wt % fat (15% linoleate and 3% α-linolenate) until 7 weeks of age when they were sacrificed [52] (Figure 2).

The rat whole body was comprised of primarily saturated fatty acids (48.4% of total fatty acids) while the monounsaturated fatty acids were present in the second greatest amount (34.8%). The total amount of *n*-6 PUFA was 12.0% and was more than 5-fold greater than the total *n*-3 PUFA. The *n*-6 PUFA with the highest content was LA (10.1%) followed by ARA (1.4%). ARA was the major PUFA in nearly every tissue and was the major PUFA in most internal organs. The tissues with the highest content of ARA were plasma (25.3%) followed by kidney, red blood cells (RBC), and spleen, ranging from 18.7% to 23.5%. Brown adipose, white adipose and the eye contained very low amounts of ARA (<1.0%). For each compartment in the rat body, the total ARA/organ was highest for muscle, then liver, adipose, and carcass. In terms of fatty acid composition expressed as a percentage, ARA was highest in the circulation, kidney, and the spleen.

As shown in rat pups, when tissues are deprived of *n*-3 PUFA, the accretion of ARA from LA is increased and ARA is further metabolized to produce docosapentaenoic acid (22:5*n*-6, DPA) which accumulates in tissues [53], particularly in the nervous system. The accumulated DPA in turn reciprocally replaces lost DHA in tissue [53].

Pigs fed varying amounts of ARA and DHA levels after birth and then sacrificed at day 28 showed that dietary ARA had little effect on tissue DHA accretion [54], but heart tissue was particularly sensitive to ARA intake. These observations are particularly notable because the pigs were fed ARA at a level of 0.53% of total fatty acids. This level is slightly above the worldwide ARA mean in human milk and 0.67% of total fatty acids is the level currently added to many infant formulas and is near the high end of human milk ARA levels [5]. Neonatal pigs serve as a practical biomedical model of human infant development due to their similar metabolic responses, genetics of the fatty acid desaturases, and rates of perinatal brain growth [5]. The importance of ARA for the heart is discussed in greater detail in Section 8.

## 4. ARA Biosynthesis and Metabolism

**Figure 3:**
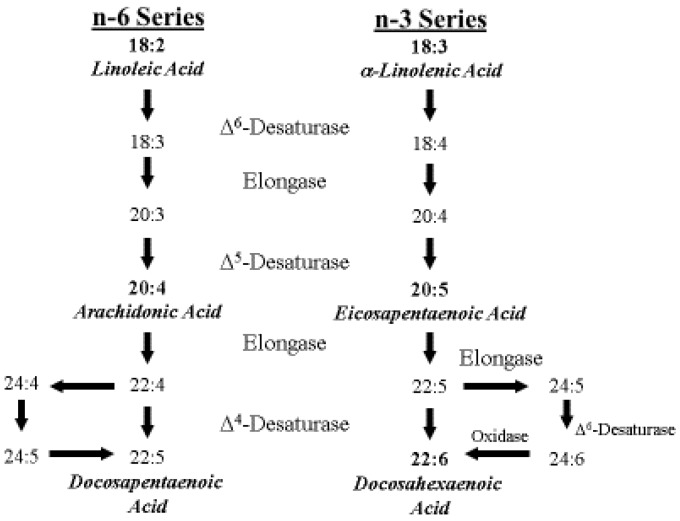
Metabolic pathways of linoleic and α-linolenic acid (Adapted from Lauritzen *et al.* [10]).

ARA is an *n*-6 polyunsaturated 20-carbon essential fatty acid formed by biosynthesis from LA [10]. ARA is a precursor to leukotrienes, prostaglandins, and thromboxanes, collectively known as eicosanoids [55,56]. ARA is found in membrane phospholipids throughout the body and is particularly abundant in the brain, muscles and liver. The metabolic pathways of the *n*-6 series and *n*-3 series are shown in Figure 3.

The use of stable isotope labelled fatty acids to investigate essential fatty acid metabolism was pioneered in the 1930s with the first identifiable study done by Schoenheimer and Rittenberg [57]. Several decades later, Nichaman *et al.* [58] gave four adult subjects ^14^C-labeled LA and found a very small but significant incorporation into plasma phospholipid ARA acid based on responses in gas-radiochromatography. Similarly, ^14^C-labeled LA was shown to be converted to ^14^C-ARA in human fetal liver microsomes, *in vitro* [59]. El Boustani *et al.* [60] studied the conversion of deuterated dihommo-gamma linoleic acid (20:3*n*-6, DGLA) into ARA in plasma phospholipid and triglyceride fractions *in vivo* in diabetic patients. After a 2 g isotope ingestion, a maximum of 5 mg of labelled ARA/L was observed in plasma. The authors stated that “this was consistent with the very low ∆5 desaturase activity observed *in vitro* in the human liver”. 

In the 1980s, Emken and colleagues [61] developed stable isotope technology in adult humans. In an early study of deuterated-LA metabolism *in vivo*, when a large dose of over 14 g of isotope was given, the authors concluded that “interconversion products such as deuterium-labeled …20:3 and 20:4 were not detected in any of the lipid classes” [62]. They calculated that a conversion of as little as 0.00012% would have been detectable. The absence of any LA metabolism to ARA was confirmed in a subsequent study even where labelled-ALA was clearly incorporated into EPA and DHA [61].

In 1995, Demmelmair *et al.* [63] used natural abundance ^13^C measurements in corn oil fed infants to demonstrate LA conversion to ARA. They observed conversion but concluded that “the activity of the enzyme system seems to be limited”. Shortly afterwards, the conversion of LA to ARA was conclusively and directly demonstrated in newborn infants using stable isotope technology by Salem *et al.* [64]. The D5-LA was used together with a highly sensitive NCI GC/MS method after PFB derivatization [65]. With this new methodology, the deuterated fatty acid could be chromatographically separated from its corresponding endogenous analogue and so the signal of the stable isotope labeled metabolite would not be obscured by the much larger signal from the endogenous fatty acid. A crude estimate was made of the net accretion of ARA over the six day period of the study which was 53 mg, or about 9 mg ARA/day. Such estimates of “net synthesis” treated the organism as if the synthesis was occurring within the bloodstream and this is clearly not the case. In addition, what was being measured is the synthesis, minus the catabolism, plus the transport/release into a compartment such as the bloodstream where it can be sampled. Carnielli *et al.* [66] used a similar methodology to confirm the conversion of LA to ARA in very low birth-weight infants using ^13^C-labeled LA.

Pawlosky *et al.* [67] studied stable isotope labeled LA and DGLA metabolism to ARA in 10 newborn human infants within the first week of life *in vivo* and performed compartmental modeling to provide an estimate of the synthetic rates. Formula and breast milk intakes were considered so that ARA and other PUFA intake could be estimated; LA and ARA intake was estimated at 3 g/kg/day and 2.8 mg/kg/day, respectively. They concluded that “the mean daily rate of synthesis and turnover of 20:4*n*-6 in plasma of infants were estimated to be from 0.06 to 2.1 mg/day…and from 0 to 51 mg/day (mean 10.2)” [67]. They went on to say that “such rates of synthesis are incapable of sustaining plasma 20:4*n*-6 concentrations in nearly all of these subjects necessitating an intake of ~4 mg/kg/day from either human milk or a supplement”. The fractional rate of conversion (FRC) observed in this study was 2.7% which is even more than that observed by Sauerwald *et al.* [68] who calculated an FSR of 0.4% to 1.1% depending upon the ALA content of the formula.

Carnielli and colleagues in 2007 [69] studied LA conversion to ARA using natural abundance ^13^C measurements in preterm infants *in vivo* in those fed LCPUFA or no LCPUFA-containing formulas at 1, 3 and 7 months of age. These authors show that ARA synthesis is decreasing with age as it fell from 26.7 mg/kg/day to 14.4 mg/kg/day and then to 11.6 mg/kg/day from 1 to 3 to 7 months of age, respectively. It seems that the endogenous synthesis rate in these infants was inadequate as the ARA plasma phospholipid level fell from 5.6 mol% in the ARA fed group to 1.9 mol% in the no ARA group, a 66% drop. This underlines the inadequacy of LA alone as a source of ARA and the requirement for preformed ARA in the infant diet if blood levels of ARA are to be maintained similar to those in breastfed infants.

## 5. Global Intake of ARA in Early Life

In contrast to *n*-3 LCPUFA, there are few data relating to dietary intakes of *n*-6 LCPUFA in early life. In relation to dietary ARA, many regulatory agencies have tended to assume that beyond the age of 6 months, the endogenous synthesis of ARA will meet the needs of infants and young children during this period of rapid growth and development [70,71]. However, studies have shown that the endogenous synthesis of both DHA and ARA may be insufficient with evidence of blood and tissue concentrations decreasing after birth if there is not an exogenous supply [49,72].

The World Health Organization (WHO) [73,74] and the American Academy of Pediatrics [75] recommend that infants should be exclusively breastfed for the first six months of life to achieve optimal growth, development and health. Thereafter, to meet their dietary requirements during growth, infants should receive nutritionally adequate and safe complementary foods while breastfeeding continues for up to two years of age or beyond [73,74]. However, there is widespread variation in compliance with this recommendation in both developed and developing countries. In an evaluation of 33 developing countries, where the health benefits of this policy could have the greatest impact, exclusive breastfeeding occurred in 46% of countries, the median duration of breastfeeding was 18.6 months and over 30% received complementary foods before 6 months of age [76]. The extent to which variation in feeding practices may influence global intakes of ARA and infant growth and development in early life needs to be further evaluated.

### 5.1. ARA Intake from Human Milk

In exclusively breastfed infants, the mean human milk intake at 6 months has been measured to be 854 g/day [73,74]. Based on those data and an estimation that 4.2% of human milk is composed of fatty acids [77] the average ARA and DHA intakes in exclusively breastfed infants at 6 months of age are about 169 mg/day and 115 mg/day, respectively. Moreover, many infants continue to receive human milk throughout the first year of life and longer. It is estimated that at 12 months of age the intake of human milk is in the range of 600–900 g/day [73,74]. This amount provides infants with an ARA intake from human milk in the range of 118–178 mg/day. The mean estimated ARA intake is approximately 12–18 mg/kg/day when adjusted for body weight using weight-for-age percentiles [78].

### 5.2. ARA Intake from Infant Formula

Infant formulas typically contain levels of ARA and DHA at 140 mg/day and 100 mg/day, respectfully, based on worldwide averages of ARA and DHA content in human milk [5]. Therefore, intakes of ARA and DHA from infant formula are similar to those provided from human milk.

### 5.3. ARA Intake from Weaning Foods

In both developed and developing countries weaning foods contain low amounts of fat, which results in a sharp transition from adequate fat intake during breastfeeding to significantly lower fat intake when children are weaned from the breast [79,80]. The main food sources of ARA are beef, poultry, eggs and seafood. Complementary foods in low-income countries are typically cereal-based and therefore LCPUFA dietary intake from these weaning foods may be minimal [79]. Countries with the lowest gross national product (GNP) (e.g., Malawi, Ethiopia, Bangladesh, Burkina Faso, Ghana and India) had a mean percentage of total PUFA from animal source foods of 4.9% *vs.* countries with a higher GNP (Vietnam, Bolivia, Indonesia, Guatemala, China, South Africa, Mexico) where the mean percentage of total PUFA from animal food sources was 18.1% [79].

Intakes of ARA (mean mg/day and estimated mean mg/kg/day) from several developing and developed countries are presented in Table 1. In the village of Keneba, Gambia, estimated mean intake of ARA during the period of 0–6 months when infants are predominantly breastfed was 90 mg/day and as complementary foods were introduced the ARA intake fell steadily to 10 mg/day at 24 months [81]. In Heqing County, Yunnan Province China, the mean intake of ARA was 55 mg/day at 1 to 3 years of age and 50 mg/day at 4 to 5 years of age [82].

Vulnerable infants and young children need energy- and nutrient-dense foods to grow and develop both physically and mentally [83]. For these reasons, dietary diversity is now included as a specific recommendation in the guidance for complementary feeding of the breastfed child aged 6 to 23 months [83]. Many factors contribute to limited dietary diversity including economic limitations, religious beliefs, and a concern that infants under 1 year of age cannot digest animal sourced foods [84,85]. There is also a widely held perception by parents that fish may be associated with allergic reactions [85,86].

Even in developed countries where dietary diversity is higher and meat and eggs contribute more to the complementary diet, the detrimental impact of the introduction of complementary feeding with low amounts of ARA and DHA content is evident. For example, mean ARA intake in German infants/toddlers decreased from 72 mg/day at 6 months of age to 24 mg/day at 9 months of age [87] (Table 1). In a separate study, these authors reported that predominately breastfed German infants had an ARA intake of 103 mg/day at 3 months of age and this amount declined to 24 mg/day at 9 months when human milk represented only 20% of the diet [88].

One hundred-seventy-four Italian breastfed children were followed from birth to 12 months of age [89]. Human milk samples were analyzed. The mean ARA intake from human milk was 95.6 mg/day at 1 month, 109.6 mg/day at 2 months, and 101.1 mg/day at 3 months. However, at 6 months of age, ARA intake sharply declined to 58.7 mg/day.

In Belgium, mean intakes of ARA were very low at 2.5 to 3 years of age and at 4 to 6.5 years of age (17 and 18 mg/day, respectively) [90] (Table 1). In Australia, national intake data indicated that 2 to 3 year-old and 4 to 7 year-old children consumed 16 mg/day and 22 mg/day of ARA, respectively [91]. Much higher mean ARA intakes were reported for Canadian children living in Vancouver where intakes ranged from 133 to 260 mg/day among children 1.5 to 5 years of age [92]. However, in children aged 4 to 7 years of age from Ontario, Canada, mean intake of ARA was lower (57 mg/day) [93].

Based on food records from the National Health and Nutrition Examination Survey (NHANES 2003–2008), the mean intake of ARA in American children at 1 to 4 years of age was 60 mg/day [94]. Most of the ARA was obtained from poultry (32.5%), eggs (27.5%), and meat dishes (20.9%). The latest NHANES data from 2015 indicate that the mean ARA intake of American children at 2 to 5 years of age increased to 80 mg/day [95].

Based on these dietary intakes from local and national surveys, it is clear that the diets of young children contain low levels of ARA. Reported mean intakes of ARA at 10 to 18 mg/day in developing and developed countries are only about 10% of the amount of ARA available to infants fed human milk or infant formulas containing DHA and ARA.

Birch *et al.* [96] reported that despite the introduction of a variety of solid foods at 17 weeks of age, infants who did not receive an infant formula supplemented with ARA and DHA throughout the first year of life had significantly lower levels of both of these fatty acids in plasma. The clinical consequences of low intake of ARA have not been adequately investigated.

## 6. ARA and Its Role in Immune System Development and Function

There is growing evidence from preclinical and clinical studies that ARA plays an important role in maintaining infant health through its effects on the immune system and through the modulation of the inflammatory response [97]. The eicosanoids that ARA produces serve as both mediators and regulators of inflammation [98] (Table 2). These immunomodulatory effects have generated much interest in the potential roles that LCPUFA in general and ARA in particular have in common inflammatory conditions in childhood such as asthma, eczema, atopic dermatitis, and food allergies [97].

**Figure 4:**
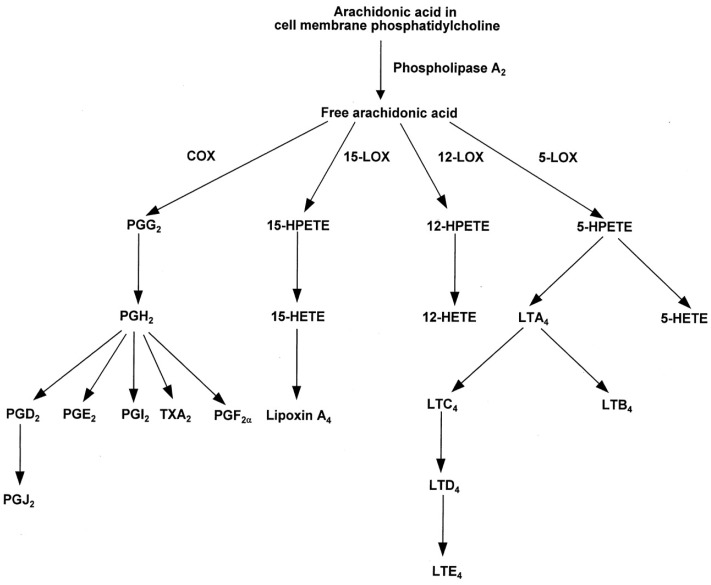
Generalized pathway for the conversion of ARA to eicosanoids. COX, cyclooxygenase; HETE, hydroxyeicosatetraenoic acid; HPETE, hydroperoxyeicosatetraenoic acid; LOX, lipoxygenase; LT, leukotriene; PG, prostaglandin; TX, thromboxane (from Calder [98]).

In cell membranes, ARA contributes to membrane order, has roles in signal transduction, and gene expression, and provides substrate for production of important chemical mediators [99]. Although ARA has been widely viewed as a pro-inflammatory agent, the eicosanoids that ARA produces serve as both mediators and regulators of inflammation [98]. ARA-derived eicosanoids, and other oxidized derivatives [98] are generated by the metabolic processes as shown in Figure 4.

Another example of the dual role of PGE_2_ and its receptors in modulating the inflammatory response has been described by Riccioti and FitzGerald [100]. During neuro-inflammation, the LPS-induced PGE_2_ synthesis causes adverse effects in neurons resulting in lesions and enhanced pain [101]. However, PGE_2_ also mediates bradykinin-induced neuroprotection by blocking LPS and ATP-induced cytokine synthesis in microglia and in neuron-glia co-cultures [102]. The anti-inflammatory and neuro-protective effects of PGE_2_ are mediated by microglial EP2- and EP4-receptors [100].

ARA is the substrate for the biosynthesis of prostaglandins. Prostaglandins and thromboxane A_2_ are collectively called prostanoids. Prostanoids are formed when ARA is released from the plasma membrane by phospholipase and metabolized by the sequential actions of prostaglandin G/H synthase, or cyclooxygenase (COX), and by respective synthesis [100]. Prostanoids serve a variety of functions. The adhesion-type prostaglandins as well as prostacyclin are important in vasodilation and in anti-thrombus formation [103]. The E series prostaglandins act to dilate arterioles and capillaries to bring about a drop in blood pressure, relax vascular smooth muscle, open the bronchi of the lungs, and enhance blood flow through the kidney [104]. Prostaglandins are also involved in sleep regulation [105], febrile response [106], and in pain perception [107].

As discussed by Calder [98], the overall physiologic (or pathophysiologic) outcome associated with the production of eicosanoids depends on the cells present, the nature of the stimulus, the timing of eicosanoid production, and the sensitivity of the target cells and tissues to the type of eicosanoids that are produced [98,108]. For example, studies have shown that prostaglandin PGE_2_, acting as a pro-inflammatory agent, induces cyclooxygenase 2 (COX-2) in fibroblast cells and by doing so up-regulates its own production [109] which in turn stimulates the production of IL-6 by macrophages (see Astudillo *et al.* [108] for a review of ARA metabolism by inflammatory cells). As an anti-inflammatory agent, PGE_2_ inhibits 5-lipoxygenase (5-LOX) thereby decreasing production of the 4-series leukotrienes [110].These 4-series leukotrienes induce 15-LOX which in turn promotes the formation of lipoxins that aid the resolution phase of inflammation [110]. Thus, the ARA-derived PGE_2_ has both pro- and anti-inflammatory effects (Table 2).

Leukotrienes, eoxins, lipoxins, and hydroperoxyeicosatetranoic acids (HPETEs) are synthesized from ARA by lipoxgenase enzymes and metabolized to LTA_4_ [108]. LTA_4_ is unstable and can be rapidly converted into LTB_4_ or LTC_4_. These three leukotrienes constitute the slow-reacting substances involved in anaphylaxis that act in allergic response [111]. They contract smooth muscle and affect vascular permeability. Eoxins are generally proinflammatory and are produced in the same manner as leukotrienes, but by the action of 15-lipoxgenase [108]. Lipoxins are produced by transcellular biosynthesis and have anti-inflammatory properties and are involved in the resolution of inflammation [112].

*In vitro* and animal studies suggest that ARA has a critical role in immune cell growth in the thymus, and in differentiation, migration, and proliferation of immune cells [98]. During early growth, there is substantial accretion of ARA in the mouse thymus which corresponds to the enrichment of the placental ARA for the fetus [98].

The immune system is composed of an integrated network of organs, tissues, cells and molecules that work together to resist infection, but maintain tolerance to harmless factors such as “self”, antigens, and allergens [113]. When a challenge is detected (e.g., an allergen or pathogen), cell signaling between immune cells produces a coordinated immune response involving the release of cytokines and eicosanoids, which under normal circumstances allows cells to communicate with each other to neutralize and eliminate the challenge [114,115].

ARA is highly abundant in platelet membranes and is closely linked to many platelet functions [116]. Due to their high numbers (*i.e.*, normal platelet count of 1.50–4.00 × 10^11^ platelets per liter of blood [117] and their ability to release inflammatory mediators, platelets perform several sentinel tasks and can quickly communicate with the cells of the immune system [118]. For example, in inflammatory skin disorders, platelets recognize bacterial pathogens through interactions with Toll-like receptors leading to the elimination of bacteria by release of antimicrobial peptides or by aggregation of platelets around the bacteria [119]. An array of receptors present on platelet membranes facilitate transduction of signals and coordinate release of chemokines, cytokines, and other inflammatory mediators to regulate inflammation and respond to invading pathogens [118,119].

Inflammation is the immune’s systems response to infection and injury [98]. Inflammation disorders are observed in infants, particularly those born prematurely [120]. Inflammation is characterized by the production of inflammatory cytokines, inflammatory agents such as reactive oxygen species, adhesion molecules, and the ARA-derived eicosanoids, and other oxidized derivatives [98]. *N*-3 LCPUFA decrease the production of the inflammatory mediators (eicosanoids, cytokines, and reactive oxygen species) and expression of adhesion molecules [98] by replacing ARA as an eicosanoid substrate and inhibiting ARA metabolism [98,121]. Aspirin and nonsteroidal anti-inflammatory drugs (NSAIDs) also inhibit the cyclooxygenase-catalyzed conversion of ARA to prostaglandins [122].

Although inflammation is perceived to be a serious health problem, the inflammation process is in fact an intrinsically beneficial event. Offending factors are removed or destroyed and as a result the affected tissues and physiological functions are restored. During the acute phase of inflammation, there is a rapid influx of blood granulocytes, typically neutrophils, followed by monocytes that mature into inflammatory macrophages [100]. The macrophages proliferate and affect the functions of resident tissue macrophages. This initial acute phase causes the usual signs of inflammation: redness, heat, swelling, and pain [100]. Once the initial adverse stimulus is removed via phagocytosis, the inflammation reaction typically decreases and ultimately resolves. During the resolution phase of inflammation, granulocytes are eliminated and macrophages and lymphocytes return to their normal pre-inflammatory levels [100]. The usual outcome of the acute inflammatory process is successful resolution and repair of tissue damage.

### Eicosanoids and Their Effects on Hormones and Bone Formation

The typical definition of a hormone is a chemical substance produced in the body that controls and regulates the activity of certain cells or organs [123]. Many hormones are secreted by special glands, such as thyroid hormone produced by the thyroid gland. Eicosanoids are recognized as different from hormones because they are not synthesized or stored in select tissues or endocrine organs. Eicosanoids are synthesized in almost all tissues and exert their biological effect near the site of their synthesis rather at a distance such as other hormones [124]. Despite these differences, eicosanoids are generally classified as hormones [125]. Eicosanoids directly affect other hormones including glycoprotein hormones. The glycoprotein hormones include luteinizing hormone, somatostatin, and glucagon. Somatostatin is an important growth hormone that controls and regulates growth and cell division [126]. It is the main hormone that stimulates cell proliferation and growth, and this hormone must be regulated so that growth is controlled [126]. Insulin and glucagon release are also affected by the eicosanoid derivatives, epoxy-eicosatrienoic acids [124].

**Figure 5:**
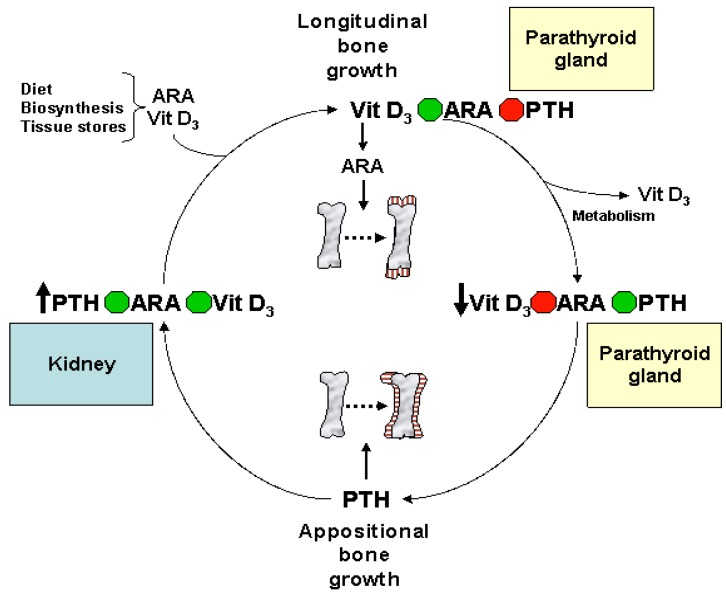
The role of ARA in bone development and homeostatic regulation of vitamin D_3_ and parathyroid hormone (PTH) levels along the parathyroid gland-kidney axis during growth. ARA and vitamin D_3_ are acquired from the diet and/or from endogenous sources. ARA mediates vitamin D_3_ regulation of chondrocyte proliferation and growth plate mineralization during bone elongation. As vitamin D_3_ is metabolized and levels subside, ARA-dependent PTH suppression is diminished and PTH production by the parathyroid gland is upregulated. This results in increased periosteal bone mineral content (appositional bone growth). In kidney, PTH induces the ARA-mediated increase in vitamin D_3_ activation and secretion, elevating the amount of vitamin D_3_ in circulation. The cycle continues as the restoration of vitamin D_3_ results in the ARA-dependent suppression of PTH and stimulates longitudinal bone growth.

ARA also plays an important role in the hormonal regulation of normal bone formation and whole body mineral metabolism during infant and childhood growth (Table 3). During skeletal development, the eicosanoids relay cellular, organ, and systemic signals to balance the calcium and phosphate needs for bone formation and other metabolic activities [127,128]. During long bone growth, when bone tissue is created, [127,128,129,130] ARA mediates vitamin D_3_-regulated chondrocyte maturation [131] and proliferation [127,132,133,134] for the mineralization of skeletal growth plates (Figure 5).

A product of ARA, prostaglandin PGE_2_ is a potent regulator of cartilage formation or chondrogenesis and resorption [135,136,137,138,139]. At low levels, PGE_2_ stimulates bone formation by increasing the production of insulin-like growth factor, a powerful growth stimulator for bone, cartilage, and muscle [140]. At high levels, PGE_2_ has the opposite effect: bone formation is reduced and resorption is increased [140].

These differential effects of osteoclast formation and resorption are mediated through multiple subtypes of G-protein coupled PGE_2_ cell surface receptors (EP1, EP2, and EP4) [141]. Activation of the EP2 and EP4 receptor subtypes are linked to an elevated level of cyclic adenosine monophosphate (cAMP) and bone formation. EP2 also acts as a selective agonist which has the ability to heal long bone fractures as demonstrated in animal models [141,142].

PGE_2_ is also critically important for bone strength [141]. When different doses (3 or 6 mg PGE2/kg/day) of prostaglandin PGE_2_ were given to Sprague-Dawley rats for 3 weeks an increase in bone and hard tissue mass, calcified cartilage cores, and a decrease in osteoclasts were observed [143]. PGE_2_ increased metaphyseal calcified tissue mass by depressing hard tissue resorption and stimulating the replication and differentiation of osteoblast precursors to form new bone [143].

Other prostaglandins play multiple roles for bone metabolism and remodeling by regulating various signaling pathways [144]. For example, PGF2α, through the activation of protein kinase C (PKC), stimulates the Na-dependent inorganic phosphate transport in osteoblasts [144]. PGF2α also up-regulates interleukin (IL-6) to stimulate osteoclast formation and increases vascular endothelial growth factor (VEGF) associated with the growth of blood vessels from pre-existing vasculature [144]. PKCα, in particular, appears to play a critical role in the regulation of osteoblastic function under load-bearing conditions [145]. During exposure to mechanical strain, PKCα is activated in osteoblast-like cells [146] while PKC signaling has been implicated in the regulation of various mechanically response genes including the osteoblast differentiation marker osteocalcin [147,148].

In studies of piglets fed formulas with differing levels of ARA (0.30%, 0.45%, 0.60% or 0.75% of fat) plus the same level of DHA (0.1% of fat), proportions of ARA in plasma, liver and adipose were dose dependent but bone modeling was not [150]. Whole-body bone mineral content was elevated in the piglets fed the highest levels of ARA (0.60% and 0.75%) and was best predicted by dietary ARA [150,154]. In addition, the 0.60% and 0.75% ARA groups had bone mineral content values closest to that of a reference group of suckled piglets [150,155].

Overall, dietary provision of ARA serves a number of important roles in skeletal metabolism. ARA functions as an important modulator of vitamin D regulation of chondrocyte proliferation and growth plate mineralization. ARA derived metabolites are important inducers of osteoclast [156,157] and osteoblast differentiation [158,159], and in modulating resorption of bone [139,149] by increasing IGF-1 gene expression [104,151] and circulating levels of IGF-1 [150]. ARA also responds to changes in stress and mechanical loading [153,160], and accelerates bone repair and healing [142,161]. Additionally, in term infants, cord blood ARA levels correlate positively with bone mineral density [155]. Thus, ARA represents an important nutrient for infant and childhood bone development and metabolism.

## 7. ARA in Skeletal and Cardiac Muscle

Several animal studies have shown that the concentration of ARA in the heart is highly sensitive to levels and ratios of ARA and DHA of the diet [24,54,162,163,164]. The amount of ARA in cardiac tissue muscle is at concentrations 2 to 3 times greater than observed in skeletal muscle [52]. Analysis of the phospholipid composition of skeletal muscle biopsies collected from 56 children <2 years of age indicated that ARA represented 16.5% of the total percentage of LCPUFA in muscle phospholipids [165].

Repetitive force loading and unloading during ATP-dependent contraction of actin filaments are major mechanical functions of heart muscle, and to a lesser extent, skeletal muscle [166]. ARA is critical for muscle contraction [166,167,168]. In skeletal muscle, excitation–contraction coupling is the process by which muscles contract [166] when a muscle action potential in the muscle fiber causes the myofibrils to contract [169]. Excitation–contraction coupling relies on a direct coupling between key proteins, the sarcoplasmic reticulum calcium release channel (the release of Ca^2+^ ions), and the voltage-gated L-type calcium channels [170]. The release of Ca^2+^ ions from the sarcoplasmic reticulum causes binding between actin and myosin to induce muscle contraction. This cycle is reset as calcium declines back to resting levels [166]. Cardiac and skeletal muscle require tight regulation of voltage-gated calcium channels and calcium homeostasis to coordinate the excitation-contraction coupling process [170].

Phosphatidylinositol (4,5) bisphosphate (PIP_2_), a phospholipid component of cell membranes, serves as an important regulator for Ca^2+^ release from the sarcoplasmic reticulum and assists in the maintenance of normal calcium signaling to control contractile forces [167,168,171]. The fatty acids of PIP_2_ are variable in different species and tissues, but studies show the most common fatty acids are stearic in position 1 and ARA in position 2 [37].

Calcium homeostasis, regulation and maintenance are critical elements for normal muscle function. Wolf *et al.* [34] have shown that the endoplasmic reticulum is directly responsible for the regulation of intracellular Ca^2+^ concentrations. ARA plays an important cooperative role with myo-inositol 1,4,5-triphosphate (IP_3_) in glucose-induced calcium mobilization and insulin secretion by pancreatic islets.

PIP_2_ and phosphatidylinositol 3,4,5-triphosphate (PIP_3_) are also critical for cardiac function [172]. In the heart, PIP_2_, as a key second messenger, controls the activity of ion channels involved with the modulation of heart rhythm. PIP_3_, on the other hand, is primarily involved in the control of cardiomyocyte apoptosis, hypertrophy, and contractility [172]. In adults, deregulation of the phosphoinositide metabolism is associated with the onset and progression of several cardiovascular pathologies including atherosclerosis and heart failure [172].

Muscle growth and atrophy depend on the balance between the rates of protein synthesis and degradation [173]. *In vitro* experiments with animal and human skeletal and cardiac muscle tissue indicate that prostaglandins are involved in the regulation of protein synthesis and degradation in various types of striated muscle [173,174]. While PGE_2_ increases degradation of muscle in young rats and causes net protein balance to become more negative, PGF_2α_ causes a large stimulation of protein synthesis in muscle tissue [173]. These findings are consistent with the many important roles played by prostaglandins PGE_2_ and PGF_2α_ in muscle protein balance and indicate that overall, ARA serves multiple functions in cardiac and skeletal muscle function and physiology.

## 8. Biomagnification and Accretion of ARA in Infants

Biomagnification is when infants have higher levels of LCPUFA in plasma lipid fractions and erythrocytes as compared with their mothers [45]. Biomagnification can be especially marked for ARA with levels more than 2-fold of that from the maternal side and independent from the amount of its precursor LA available maternally. The stability of LA content implies that any conversion to ARA is not keeping up with the fetal demand for ARA [45]. Biomagnification by the placenta serves to preferentially obtain preformed ARA and DHA from the mother in order to deliver it to and nourish the fetus [45].

As shown by Kuipers *et al.* [175], biomagnification is independent of maternal ARA status at both delivery and at 3 months of age and is found to be similar across different populations with differing diets. These findings indicate that biomagnification as a biological process seeks to achieve a uniform ARA status in infants at the expense of their mothers. The process of biomagnification suggests that a certain level of infant prenatal ARA status must be maintained for optimal infant growth.

Infants with the lowest birthweights have the lowest levels of ARA, and those born earliest have the lowest levels of DHA [45]. The process of biomagnification initially protects vascular growth which is a requirement for brain growth. Vascular growth must precede brain growth to meet the brain’s demand for energy, which can be as high as 70% of the total fetal demand for energy in the last trimester of pregnancy [45].

At delivery, as shown by Luxwolda *et al.* [176], the maternal RBC-ARA content is consistently higher than that at 3 months postpartum. At delivery, infant RBC-ARA content is similar or higher than their mother’s RBC-ARA contents. From delivery to 3 months postpartum, maternal RBC-ARA increases while infant RBC-ARA decreases. The decrease in RBC-ARA content may be due to a lower conversion of LA to ARA since the infant’s capacity to synthesize LCPUFA decreases dramatically after delivery [69] and has been shown to decrease with gestational age at birth [64].

There appears to be a tightly regulated synergism between DHA and ARA at low DHA status and an antagonism at high DHA status [50]. Intrauterine DHA biomagnification in mothers with low fish intakes aims at a synergistic increase of fetal DHA to maintain a balance with ARA. Bioattenuation at higher DHA status may in turn prevent abundant passage of DHA across the placenta that leads to antagonism with ARA. Since ARA is important for fetal growth [177] and is rapidly accreted in the fetal brain [178,179] any competition from gestational DHA must be tightly regulated and balanced for optimal neurodevelopment after birth [50]. Dietary depletion of ARA in early infancy may have adverse consequences for brain development [178,179].

## 9. Consequences of ARA Deficiency

Essential fatty acid (EFA) deficiency impairs lipid and energy metabolism, cell membrane structures, lipid signaling pathways, and ultimately leads to death [180,181]. Mammals are dependent on a dietary supply of LA and ALA which are converted into *n*-6 and *n*-3 PUFA, respectively. Δ6-fatty acid desaturase (FADS2) converts LA to γ-linolenic acid (C18:3*n*-6) and Δ5-fatty acid desaturase (FADS1) converts dihommo-γ-linolenic acid (C20:3*n*-6) to ARA [182].

Early studies of EFA deficiency considered the effect that various dose levels of intake of LA, ARA and ALA esters (0% to 10% of total calories for 100 days) had on the fatty acid composition in the liver of rats [183]. Fat deficiency symptoms (necrotic tail and scaly feet) appeared in all animals fed LA at less than 0.6% of calories and ARA at less than 0.25% of calories. ARA was 3-fold more effective than LA in liver incorporation and mitigating deficiency. Fat deficiency symptoms affecting the skin were not surprising. In the skin, as in all organs, EFA are principally found in glycolipids and phospholipids. Most of the epidermal fatty acid PUFA is ARA [184]. EFA deficiency causes skin flaking in humans, dogs, and mice, symptoms that can be restored with LA dietary therapy [184].

Since LA deficiency results in disruption of the skin’s water barrier function [185] and heat loss from skin [186] the side effects make it difficult to distinguish the specific effects of ARA deficiency independent from those related to LA deficiency. The fads2^−/−^ mouse allows for the specific investigation of ARA deficiency without the underlying complications of LA deficiency [181]. The mutation eliminates Δ-6 desaturase activity leading to a dramatic decrease in the accumulation of ARA in tissues and subsequently, ARA conversion to PGE, TXB, prostacyclin and leukotrienes. Platelet aggregation and thrombosis are therefore also limited.

When fads2^−/−^ mice were followed for several weeks and fed a diet lacking Δ6-fatty acid desaturase products but containing ample amounts of LA, the lack of PUFA and eicosanoids did not impair lifespan but all the mice were sterile, developed ulcerative dermatitis, splenomegaly, and ulceration in the duodenum and ileocecal junction [182]. Liver levels of ARA and DHA declined by 95% and somewhat smaller decreases were observed in the brain and testes (~50%). The absence of γ-linolenic conversion in the fads2^−/−^ mouse deprived the cyclooxygenase and lipoxygenase pathways of their substrates, including the elimination of PGE synthesis, the failure of synthesis of TXBs by thrombocytes, and the failure to produce leukotrienes [181]. PUFA supplementation completely restored the adverse symptoms observed in fads2^−/−^ mice. The mechanism by which ARA prevented dermatitis may be due, at least in part, to lower levels of prostaglandin D_2_ (PGD_2_) when skin ARA is decreased [182].

The ulceration of the small intestine in the fads2^−/−^ mouse may have been associated with the decline of prostaglandin synthesis, similar to the effect often seen with the long-term use of NSAIDs [187]. NSAIDs block prostaglandin synthesis by inhibiting cyclooxygenases, leading to an erosion and then ulceration of the mucosal layer of the stomach and small intestine. However, loss of organized stratification of proliferating cells into defined zones is a common feature of EFA deficiency [188].

The Δ6-fatty acid desaturase gene FADS2 was cloned in 1999 [189]. An adult human case of Δ6-fatty acid desaturase deficiency was identified and described in the literature [190]. The patient exhibited growth retardation accompanied by skin abnormalities, corneal ulceration, and feeding intolerance. Treatment with dietary DHA and ARA restored normal growth and eliminated most of the symptoms [190].

**Figure 6:**
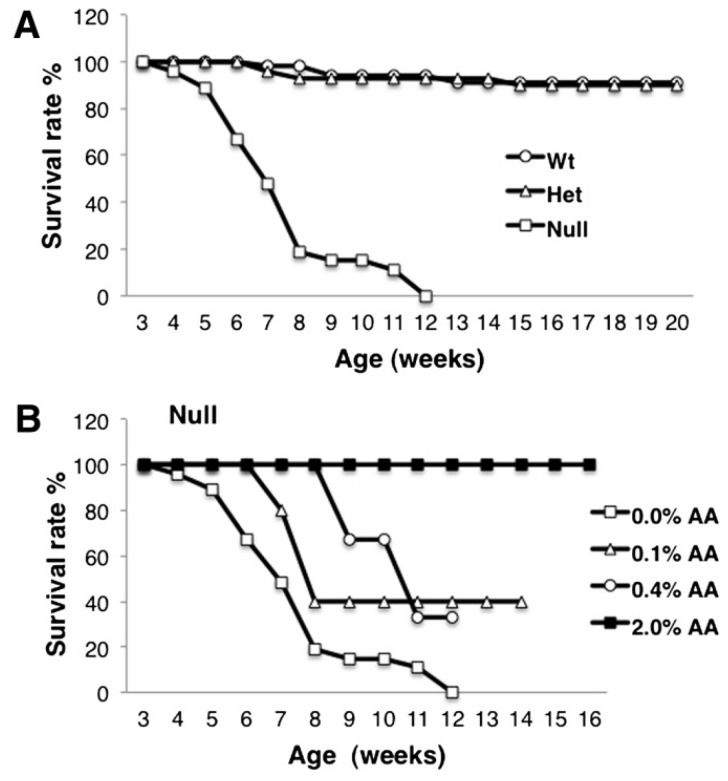
Kaplan-Meier survival curves of *Fads1* mice, AA = ARA. (**A**) *Fads1* null mice exhibited low viability when fed a standard AA-free diet; *n* = 37 for wild-type, *n* = 44 for heterozygous, *n* = 11 for Null; (**B**) Dietary supplementation with AA (0.1% and 0.4%, *w*/*w*) partially reversed the *Fads1* null mouse phenotype; *n* = 5 for Null + 0.1% AA, *n* = 3 for Null + 0.4% AA. Supplementation with 2.0% AA completely reverse the Null phenotype; *n* = 4 for Null ± 2% AA (from Fan *et al.* [191]).

A novel genetic model, the FADS1 (Δ5 desaturase) knockout mouse was used to determine the role that the ARA-derived 2-series eicosanoids had in mucosal physiology and inflammation [191]. Fads1^−/−^ mice have very low levels of ARA in tissues (colon mucosa, liver, spleen, serum and fatty acid profiles). The deficiency in ARA resulted in a massive enhancement of dihomo-y-linolenic acid, the 1-series prostaglandin substrate in tissues and a decrease in 2-series-derived prostaglandins or PGE_2_. Fads1^−/−^ mice failed to thrive, gradually dying at 5 to 6 weeks of age with no survivors past 12 weeks of age [191]. The lack of PGE_2_ was associated with disturbed intestinal crypt proliferation, altered immune cell homeostasis, and a heightened sensitivity to acute inflammatory challenge [191]. Dietary supplementation with ARA extended the longevity of fads1^−/−^ mice to levels comparable with normal wild-type mice (Figure 6).

Although fads1^−/−^ and fad2^−/−^ mice are useful to examine the function of ARA *in vivo* PUFA are transferred through the placenta from the heterozygous mother into the homozygous fetus. Additionally, the amount of DHA and ARA in the brain tends to remain tightly controlled even under conditions of PUFA deprivation, but at the expense of other tissues to protect the brain [182]. Lpiat1^−/−^ mice have a mutation that affects the synthesis of ARA-containing PI. PI is unique in its fatty acid composition, *i.e.*, most of the fatty acid that is attached to the sn-2 position of PI is ARA [33]. Other membrane phospholipids such as PC and PE contain other PUFA including DHA.

Lee *et al.* [33] showed that Lpiat1^−/−^ mice had a reduced content of ARA in PI and had deficits in cortical lamination during brain development, delayed neuronal processes in the cortex, and reduced neurite outgrowth *in vitro*. Lpiat1^−/−^ mice died within a month and showed atrophy of the cerebral cortex and hippocampus. These results demonstrate the importance of ARA-containing PI in normal cortical development in mice. By eliminating LPIAT1 in Lpiat1^−/−^ mice, the enzyme responsible for the incorporation of ARA into PI, it was shown that the ARA-containing PI is essential for brain development in mammals [33].

Newborn pups of Δ6-fatty acid desaturase knockout mice were administered artificial milks that contained 3.7% ALA and 16% LA with or without 1.2% ARA and/or 1.2% DHA for 18 days immediately after birth [192]. Compared with wild-type mice, the body weight of the mice fed the control diet was significantly lower, particularly after 6 weeks of age. However, body weights of knockout mice fed milks with DHA and ARA+DHA were similar to that of the wild-type mice. Motor activities of the knockout mice fed ARA were elevated compared with the wild-type mice and those fed the control diet. Better motor performance was also observed in knockout mice fed the ARA + DHA diet. The authors concluded that ARA corrected the decrease in body weight and the combination of ARA and DHA improved the motor dysfunction caused by the deficit of Δ6-fatty acid desaturase.

Taken together, results from these investigations indicate the importance of ARA and its derivatives for the coordination of cellular differentiation, organogenesis, and function during early growth and development.

## 10. Animal Studies of ARA Supplementation

### 10.1. Immunomodulatory Effects of ARA and DHA Supplementation

**Figure 7:**
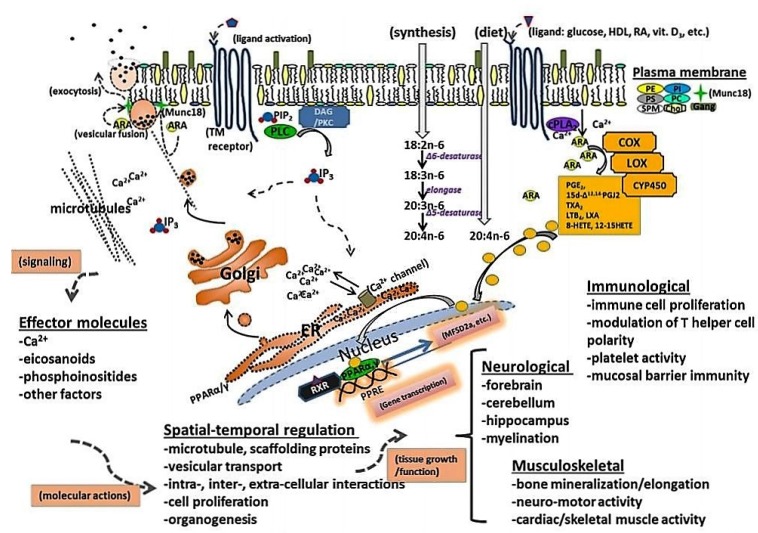
Schematic summary of molecular events and functional outcomes involved in metabolism of ARA. ARA is derived from endogenous synthesis or directly from the diet and is incorporated into cellular membrane complex lipids. Within the lipid bilayer, ARA is enriched in PE and PI in the inner membrane. Coordination of spatial-temporal interactions between molecular and cellular components and activities are mediated by metabolites of, or molecules associated with metabolism of ARA. Metabolism of ARA is triggered by activation of transmembrane receptors as a result of binding a ligand. A few examples of receptor-mediated activation of ARA metabolism include glucose, vitamin D_3_, Ca^2+^, or antigen presentation or detection by immune cells. ARA released from the membrane by the actions of PLA_2_ or metabolized by enzymes such as COX, CYP450, and/or LOX can act directly or serve as a substrate for various enzymes to produce second-messengers. ARA-derived eicosanoids, including prostaglandins, leukotrienes, lipoxins, and HETEs regulate numerous activities including passage of ions between subcellular compartments, interactions between various structures or cells, and nuclear regulation of gene transcription by PPARs activators. Within the inner leaflet of cell membranes, ARA is enriched in micro-domains and is involved in regulation of receptor mediated activities. In addition, micro-domains serve as foundations for biophysical interactions between subcellular structures such as microtubules and other cytoskeletal activities including vesicular transport. The consequences of temporal-spatial regulation include coordinated release of hormones, expression of various cell functions, and/or alterations in phenotypes, and cellular motility. Examples of PPAR-regulated gene products involved tissue uptake of LCPUFA and oxidation of stored lipids: MFSD2A, major facilitator of superfamily domain-containing protein 2A. Membrane components: Chol, cholesterol; Gang, gangliosides; PC, phosphatidylcholine; PE, phosphatidylethanolamine; PI, phosphatidylinositol; PS, phosphatidylserine; SPM, Sphingomyelin. Nuclear transcription factors: PPAR, peroxisome-proliferator activator receptors; RXR, retinoid X receptors. TM, transmembrane receptors. Enzymes: COX, cyclooxygenase; CYP450, cytochrome P450; LOX, lipoxygenase; PLA_2_, Phospholipase A2; PLC, Phospholipase C. Signaling molecules: PG, prostaglandin (Adapted from Pike [32]).

The activation of peroxisomal proliferator-activated receptors (PPARs) has been shown to be protective in brain ischemic and oxidative injury and in many neurological diseases that may affect infants [193] (Figure 7). In addition, transcription of the gene for the Major Facilitator Superfamily of the domain-containing protein 2a (MFSD2A) has been identified as being an important transporter for the uptake of DHA across the blood brain barrier [194] and is under the control of PPAR [195]. Studies indicate that LCPUFA and their metabolites are ligands to PPARs. Diets containing an *n*-6:*n*-3 ratio of about 1-2:1 supplied during pregnancy and lactation appear to be optimal for the expression of neuron-specific enolase, glial fibrillary acidic protein and myelin basic protein, markers related to the growth and maturation of neurons, astrocytes and myelin [193].

To investigate the immunomodulatory effects of different PUFA, weanling rats were fed a high-fat diet (178 g/kg) that contained 4.4 g of ALA, γ-linolenic, ARA, EPA, or DHA/100 g total diet [196] for 6 weeks. The proportion of total PUFA content (~35 wt %) was held constant and the *n*-6 to *n*-3 ratio was maintained at 5.8 to 7.0. PGE_2_ production was enhanced in leukocytes from rats fed the ARA-rich diet and was decreased from leukocytes in rats fed the EPA or DHA diets. ARA did not affect lymphocyte proliferation, NK cell activity, or the cell-mediated immune response. Lack of an effect on T-lymphocyte proliferation and Con A in splenocyte cultures was also observed in mice fed a safflower oil ethyl ester diet +1% ARA for 10 days [197]. The lack of an immunological effect of ARA agrees with findings from a human study that considered 1.5 g of ARA/day for 50 days on the proliferation response of peripheral blood mononuclear cells to Con A, phytohemagglutinin, or poke-weed mitogen [198]. Human peripheral blood NK activity was also unaffected by the consumption of ARA.

Prostaglandins which are involved in inflammatory processes also play a major role in the recovery of intestinal barrier function in ischemia-injured porcine ileum by converting ARA to PGH_2_ [199]. The importance of ARA and ARA-derived eicosanoids in the intestinal epithelium was reviewed by Ferrer and Moreno [200]. In a study that considered the effect of supplemental ARA on intestinal barrier repair in ischemia-injured porcine ileum pigs were fed a formula containing no LCPUFA (0% ARA), 0.5% ARA, 5% ARA, or 5% EPA for 10 days. Piglets that were fed 5% ARA exhibited enhanced recovery compared with piglets fed 0% ARA or 0.5% ARA [201]. The EPA-fed piglets had enhanced recovery comparable with piglets fed 0% ARA. The enhanced recovery response observed with 5% ARA was supported by reduction in the mucosal-to-serosal flux of ^3^H-mannitol and ^14^C-inulin compared with the other dietary groups. Jacobi *et al.* [201] concluded that piglets fed a high-ARA diet are less susceptible to ischemia-induced epithelial cell sloughing and that feeding elevated levels of LCPUFA, including ARA, enhances acute recovery of ischemia-injured porcine ileum. For infants affected by necrotizing enterocolitis (NEC) where physiological repair of the intestines is necessary elevated LCPUFA intake including ARA enhances recovery of damaged tissues [201].

EFA deficiency also leads to hepatic steatosis. When rats were administered varying amounts of DHA and ARA to determine whether exclusive supplementation with DHA or ARA could prevent EFA deficiency and inhibit the development of hepatic steatosis mice fed at least 2% of their calories from DHA and 1% of the calories from ARA did not develop clinical or biochemical evidence of EFA deficiency disease or hepatic steatosis [202]. Although hepatic steatosis is an adult disease, the fact that mice fed at least 2% of their calories from DHA and 1% of the calories from ARA prevent the development of EFA deficiency suggests the importance of ARA throughout the lifespan.

To investigate the ability of ARA- and ARA + DHA-enriched formula to modulate immune response in neonatal piglets to an inactivated influenza virus vaccine Bassaganya-Riera *et al.* [203] considered a diet with ARA + DHA in sow milk fed at birth. The diet modulated antigen-specific T-cell responses to an inactivated influenza virus and up-regulated IL-10 expression [203]. Although ARA and DHA have been suggested to elicit opposing immunomodulatory actions, the immunologic outcome in the study was beneficial [203]. The authors concluded that ARA + DHA enriched formulas, with the approximate 2:1 ratio fed during the neonatal period, may prevent or manage autoimmune and allergic reactions in infants by down-modulating T-cell reactions.

### 10.2. Retinal and Neurodevelopmental Effects of ARA and DHA Supplementation

The retinal DHA content in guinea pigs was considered in relation to diets containing different *n*-6:*n*-3 ratios (from 72.0 to 2.5) [204]. Not surprisingly, diets with the highest *n*-6:*n*-3 PUFA ratios had the highest *n*-6 retinal fatty acid profiles. Weisinger *et al.* [204] reported that retinal function was altered by tissue DHA levels and responded according to an inverted “U-shaped” function. As DHA levels increased past an optimal amount found to be 19%, the result was poorer electroretinographic scores. However, there was no mention that as DHA increased there was a corresponding decrease in ARA levels due to ARA antagonism. The marked decrease in ARA levels may have been the variable of interest that was not fully considered and responsible for the electroretinographic changes at high DHA intakes.

Champoux *et al.* [205] used a neurodevelopmental battery to test the neurological behavior in rhesus macaques neonates fed a control formula without LCPUFA or a LCPUFA-supplemented formula with 1 wt % each of DHA and ARA. Macaque neonates fed the supplemented formula obtained higher scores on motor maturity and orientation than those fed a control formula. Champoux *et al.* [205] concluded that the results supported the view that preformed DHA and ARA in infant formulas are required for optimal neurological development.

Learning behavior in rats fed a diet supplemented with 3% safflower oil (Safflower, *n*-3 fatty acid deficient, high LA acid) was compared to those fed 3% perilla oil (Perilla, high ALA) [206]. Through two generations, the *n*-3 fatty acid deficient group exhibited decreased correct response ratios in a brightness-discrimination behavior test. The altered learning ability in the brightness-discrimination test was restored with supplementation of DHA after weaning, only after levels of ARA in the brain lipids were normalized. The authors concluded that *n*-3 fatty acid is essential for the maintenance of learning performance and that *n*-3 deficiency in the presence of *n*-6 fatty acid during gestation did not lead to irreversible damage to the brain [206]. Thus, both DHA and ARA affected learning performance and a balance of ARA and DHA levels must be maintained.

To investigate the effects that varying dietary levels of LCPUFA have on growth, brain fatty acid composition and behavior in mice, 5 groups of pregnant and lactating mice were fed diets with either very high *n*-6 to *n*-3 ratio of 49 (*n*-3 deficient), a more usual ratio of 4.0, or a low ratio of 0.32 for 15 weeks [207]. There was no effect of diet on birth weights of pups, but on days 15 and 22 the pups in the low *n*-6 to *n*-3 groups weighed less than those in the other treatment groups. Increasing levels of DHA in the diet increased brain DHA and decreased brain ARA. The differing ratios of *n*-6 to *n*-3 had no effect on the ability of mice to learn the place test or perform in the Morris water maze. However, the mice fed the low *n*-6 to *n*-3 ratio swam more slowly, unless ARA was substituted for LA as the source of the *n*-6. The lower body weight in the high *n*-6 to *n*-3 fed mice was not attributed to simply *n*-6 deficiency. The high *n*-3 to *n*-6 ratio led to the inhibition of Δ6-desaturase [207]. Thus, the conversion of LA to ARA was impeded and ARA became unavailable for growth. Mice fed high levels of DHA also had high levels of EPA showing a considerable amount of retroconversion. The findings showed the importance of balancing the amounts of ARA to DHA and that some deficits can be overcome if LA is replaced by ARA as the source of *n*-6 fatty acid [207]. Wainright *et al.* [208] also reported that ARA supplementation increased ARA levels and decreased DHA levels in forebrain membrane phospholipids in Long-Evans rats, whereas DHA supplementation increased DHA levels and decreased ARA levels. Correlational analyses did not show a relationship between DHA and ARA levels in the forebrain and working memory performance [208,209].

Newborn infants of diabetic mothers have lower ARA and DHA levels in cord blood than newborns of normal mothers [210]. The lower levels of the LCPUFA in the newborns of diabetic mothers were associated with impaired and altered sensory-cognitive and psychomotor functions at birth and reduced visual and memory performance at 8 and 12 months [211,212]. Compared with normal controls, most rat models of diabetes are characterized by a lower level of brain ARA only and not a lower level of DHA [213]. Even though both ARA and DHA are important for neurodevelopment, brain accretion of ARA exceeds that of DHA during gestation [214], especially in the first two trimesters during the period of rapid proliferation of neuronal and glial elements [179,215]. When Sprague-Dawley diabetic, pregnant rats were fed either a control diet or an ARA (0.5%) supplemented diet throughout reproduction, the weaned offspring in the ARA group performed significantly better in the water maze and rotarod tests and showed greater exploratory behavior than control-diet offspring [216]. The results indicated that maternal hyperglycemia has long-term consequences during the initial stages of learning and that maternal supplementation with ARA positively influences learning outcomes.

Amusquivar *et al.* [217] reported that rat pups of dams fed diets with *n*-3 fatty acids from fish oil compared with those fed *n*-6 fatty acids from olive oil during pregnancy and lactation had smaller increases in postnatal body weight and length, and delayed body and psychomotor maturation indices. Slower growth and brain development occurred when both dams and fetuses were fed a moderate amount of fish oil (10%) as the only fat source [217]. In the study, the ARA level was lower than the DHA level in brain tissue of the offspring of dams fed high *n*-3 fatty acid diets. The differences in postnatal development disappeared when the fish oil was supplemented with γ-linolenic acid, a precursor of ARA. The growth deficits were also eliminated by the inclusion of ARA in the diet [207]. The studies demonstrate the importance of maintaining adequate levels of ARA during development, and suggest that diets too high in *n*-3 fatty acids during development may have negative effects on development by reducing tissue levels of ARA [218].

To investigate the effects that a DHA-rich maternal diet compared with an ARA-only diet have on brain fatty acid composition of Sprague-Dawley rats, Elsherbiny *et al.* [219] considered a control diet containing ARA (0.4 g/100 g of total fatty acid) *vs.* a DHA + ARA diet (0.9 g/100 of DHA and 0.4 g/100 g of ARA of total fatty acid). The results indicated that at three weeks postnatally the DHA-rich diet increased levels of DHA in the brain and decreased ARA by 12.8%. The brain of a three-week-old rat is at a comparable stage as that of a human toddler at 2–3 years of age [219]. At six weeks (comparable to a 12–18 years old human), the DHA-induced decreases in ARA were reversed and disappeared when DHA was continued (*i.e.*, DHA/control group). Thus, elevated dietary levels of DHA decrease the amount of ARA in brain without an adequate supply of dietary ARA.

Prepulse inhibition (PPI) is a normal suppression of a startle response when a low intensity stimulus that elicits little or no behavioral response immediately precedes an unexpected stronger startling response [220]. Deficits in PPI have been reported in individuals that have mental disorders including schizophrenia [220]. Various brain regions including the hippocampus have been associated with PPI problems. To determine whether dietary administration of LCPUFA enhances neurogenesis in the rat hippocampus and improves PPI response in wild-type mice and Pax6^+/−^ mice (that exhibit PPI deficits) a control diet or diets supplemented with ARA (4%), DHA (4%) or ARA (4%) + DHA (4%) were administered [220]. Compared with the other diets, the administration of the ARA diet successfully increased neurogenesis not only in the Pax6^+/*−*^ mice but also in the wild-type mice. Treating the Pax6^+/*−*^ mice with ARA also resulted in alleviating their PPI deficits. The authors suggested that the ARA diet as compared with the DHA or ARA + DHA diets positively affected postnatal neurogenesis in several regions of the brain including the hippocampus by influencing the fluidity of neuronal membranes and by regulating neuronal transmission [220].

## 11. Introduction of DHA and ARA in Infant Formulas

Both DHA and ARA have been added to infant formulas in the United States since 2001. In Europe, the addition of DHA and ARA in infant formulas began much earlier in 1994. Most infant formulas contain 0.2% to 0.4% total fatty acids of DHA and between 0.35% and 0.7% total fatty acids of ARA based on worldwide averages of DHA and ARA content in human milk [5]. Several international expert groups [6,7,8,9] support the addition of these levels of DHA and ARA in infant formulas to ensure optimal infant growth and development. Thus, all commercially available infant formulas contain preformed ARA at levels equal to or higher than the DHA content in formulas where these LCPUFA are added.

To determine the necessity of adding LCPUFA to infant formula, several studies were performed in the 1990s with preterm and term infants fed formulas containing DHA or EPA with and without ARA (see Fleith and Clandinin [221] for a review). No studies have examined supplementation of infant formula with ARA alone. In most studies, a control group without LCPUFA and/or a breastfed group were included. Studies also investigated the effect of adding ALA to ensure a sufficient endogenous synthesis of DHA [222,223], but not surprisingly, due to the limited conversion of ALA to DHA, the added ALA was not effective in raising DHA plasma status to the same level as that observed in breastfed infants ([223]. Some studies also considered experimental formulas containing added γ-linolenic acid from black current-seed oil, borage oil, or evening primrose oil, DHA and EPA from marine oil, and ARA from egg phospholipids [223,224,225,226,227,228,229]. The effects of feeding formula supplemented with soy oil and marine oils containing DHA/EPA showed no abnormalities on growth, tolerance, clotting function, erythrocyte membrane fluidity and vitamin A or E levels in low-birth-weight-term infants [230].

Infant formulas containing DHA and ARA from single cell oils (DHASCO^®^ and ARASCO^®^, DSM, Columbia, MD, USA), respectively, were evaluated and found to maintain both DHA and ARA status in infants [178,231,232,233]. After 2001, DHASCO and ARASCO (DSM, Columbia, MD, USA) became predominant as the sources of DHA and ARA added to infant formulas in the United States. Both DHASCO and ARASCO are general recognized as safe (GRAS) for use in infant formulas in the United States and approved as novel foods in Canada [234,235]. DHASCO and ARASCO have an established history of use in Europe, Australia and New Zealand and are not considered novel foods and can be added to infant formulas (see Ryan *et al.* [236] for a review of the safety of single-cell oils).

The clinical studies used to evaluate the effects of DHA and ARA added to infant formulas measured infant growth, body and fatty acid composition, behavioral and sensory functions (retinal function, visual acuity and auditory function). Many of the early studies focused on preterm infants because they provided an opportunity to evaluate the effects of DHA- and ARA-enriched formulas in infants who may be deficient in these LCPUFA. Transfer across the placenta and accretion of ARA and DHA in the developing human brain and retina occurs mainly during the last trimester of pregnancy [179,215] when the rate of brain growth is most rapid [237]. Infants born prematurely thus may have an increased need for dietary ARA due to the interrupted supply during the last trimester [220,238,239].

Four studies with preterm infants considered formulas without added preformed ARA [240,241,242,243]. Each was influential in recognizing the importance of ARA for optimal growth. Two of the studies reported an increase in visual acuity at 2 or 4 months postmenstrual age (PMA) in preterm infants fed formulas supplemented with DHA and EPA from fish oil with a low or high ratio of DHA to EPA (2:1, 5:1, respectively) and no ARA [241,244,245,246].

In one study [240], infants fed formula with a low ratio of DHA to EPA until 79 weeks PMA, compared with controls, had significantly lower z-score values for weight, length and head circumference beginning at 40 weeks PMA. Poorer growth was also associated with lower scores of psychomotor development [244]. The supplemented group also had lower blood levels of ARA [177] suggesting that the effects of growth may have been related to the reduced availability of ARA as a result of the competitive inhibition by the high levels of EPA in marine oil. This finding was supported in a second study [241] in which preterm infants were fed formula with a high DHA to EPA ratio until 48 weeks PMA. Compared with controls, preterm infants fed a high ratio of DHA to EPA and with no ARA consistently had lower mean weight-for-length values at 2, 6, 9, and 12 months PMA, weighed less at 6 and 9 months PMA, and had smaller head circumferences at 9 months PMA [246].

In a third study, three premature infant formulas were compared in a double-blind parallel-group study of the growth of healthy, very-low-birth-weight infants (846–1560 g at birth) [242]. The DHA formula contained 0.34% of fat as DHA and the DHA +ARA formula contained 0.33% as DHA and 0.60% as ARA. The control formula contained no DHA or ARA. A reference group consisted of term infants who were predominately breastfed. Results indicated that infants fed formula with DHA + ARA gained significantly more weight than infants fed formula without DHA+ARA. At 48 and 57 weeks, weight of infants in the DHA + ARA group did not differ from the reference group of term infants. Length of infants in the DHA + ARA group was significantly greater than that of infants fed DHA alone at 40 and 48 weeks, but not at 57 weeks. The authors concluded that supplementation with ARA in addition to DHA supported growth of preterm infants [242].

In a fourth study [243], male but not female preterm infants fed formula with DHA and EPA from fish oil (0.2 wt %, 5:1 ratio) and no ARA, or a control formula to 59 weeks PMA, had significantly smaller gains in weight, length, and head circumference and lower fat-free mass as determined from total body electrical conductivity (TOBEC).

In the Carlson *et al.* [177,240] studies that reported slower growth in preterm infants a positive association between plasma ARA concentration and measures of growth (weight, length, z-score, weight-to-length, and head circumference) was observed. In the 1990s, when the studies were conducted, it was unknown whether the negative effects of DHA and/or EPA supplementation on growth could be overcome by adding ARA to infant formulas [177]. However, it was known that the bioactive metabolites of ARA mediate the secretion of several hormones associated with growth and basic metabolic functions [247]. These include luteinizing hormone, prolactin, adrenocorticotropic hormone (ATCH), and corticotropin-releasing hormone (CRH) [241] which could influence growth. ARA and its second messengers also appear to be involved in bone formation and resorption [248].

To maintain ARA status in preterm infants, additional studies were performed with infant formulas containing both ARA and DHA [221]. The addition of ARA at levels found in breast milk produced growth comparable to that observed in breastfed infants [221]. Adding both ARA and DHA to preterm infant formulas also resulted in beneficial effects on visual acuity as compared with infants fed a control formula [221].

Concern with a high level of DHA without a concomitant increase of ARA was raised in a randomized, controlled clinical trial of term infants administered formula with no LCPUFA, or differing levels of DHA intakes of 0.32%, 0.64% and 0.96% at the same ARA level of 0.64% [249,250]. There were no formula effects on tests of behavioral and psychophysiological indices of attention at 4, 6, and 9 months of age. However, infants supplemented at the two lower doses of DHA spent proportionately more time engaged in active stimulating processes (increased attention) than infants in the unsupplemented group [249]. Positive results were also observed on vocabulary (Peabody Picture Vocabulary), a card-shorting task, and an intelligence test (Wechler Primary Preschool Scales of Intelligence) at 3 to 6 years of age in the two lower doses of DHA (0.32% and 0.64%) [250]. However, performance of infants and children who were administered the highest dose of DHA (0.96%) but with a reduced ratio of ARA to DHA was attenuated [250]. The results demonstrated that a proper balance of DHA and ARA is needed for optimal cognitive performance as too much DHA may suppress the benefits provided by ARA.

The effects that different ratios of *n*-6 to *n*-3 had on preterm infant neurodevelopment were recently considered by Alshweki *et al.* [251]. Preterm infants (<1500 g and/or <32 weeks gestational age) were given infant formula with an *n*-6 to *n*-3 ratio of 2:1 or 1:1. The infants were followed for up to 2 years. Preterm infants fed formula with a 2:1 ratio of ARA to DHA had higher ARA blood levels during the first year of life and better psychomotor development compared with those fed a 1:1 ratio of ARA to DHA. However, despite the fact that one group received twice the amount of ARA than DHA (66% *vs.* 33%), there was almost no difference between the two groups in plasma *n*-6 to *n*-3 ratio. The balance between *n*-6 and *n*-3 is very complex, but appears to be maintained at a steady level when adequate supplementation of ARA and DHA are available [251].

To date, several systematic reviews of the literature and meta-analyses have been published to evaluate the effects of LCPUFA in preterm and term infants [221,229,252] on various outcomes including growth, cognition and vision. The reviews considered both the earlier and more recent studies on LCPUFA. Fleith and Clandinin [221], in one of the earliest reviews, reported that collectively the body of literature supported the view that LCPUFA are important for growth and development of preterm and term infants. Formula levels of ARA and DHA should be in the same range as those found in human milk and with the same ratio [221]. There needs to be a dietary supply of ARA and DHA to achieve similar accretion levels in plasma and in RBC as compared with breastfed infants [221,253,254].

A Cochrane review of LCPUFA supplementation in term infants reported that there was little evidence that supplementation conferred a benefit on visual or cognitive development [229]. In a recent meta-analysis of four clinical trials including data for preterm and term infants, LCPUFA supplementation was also shown to have no effect on Bailey Developmental scores at 18 months of age [252]. However, as pointed out by Colombo *et al.* [250], the lack of an observed effect at 18 months of age is consistent with the view that the Bailey Scales of Infant Development are not very sensitive to the effects of LCPUFA supplementation. The Bailey Scales of Infant Development yield a composite score obtained from the infant’s attainment of normal developmental milestones and may not be able to provide detailed assays of specific cognitive mechanisms that are measured using more sophisticated laboratory tasks [250]. This raises the question of whether the Bailey Scales are appropriate for measuring the effects of LCPUFA on cognitive development in older infants/toddlers.

A systematic review of 20 randomized, controlled trials of term infants who received DHA and ARA supplemented formula or a control formula indicated that infants given formulas containing DHA levels close to the worldwide human milk mean of 0.32% of total fatty acids were more likely to yield positive results on cognitive and visual tests [255]. There was also clinical evidence to suggest than an ARA:DHA ratio greater than 1:1 was associated with improved cognitive outcomes [255].

Since the publication of the Hoffman *et al.* [255] review, several epidemiological and interventional studies of LCPUFA supplementation during infancy have appeared in the literature. These recent publications have been reviewed by Ryan *et al.* [256]. The most recent data indicate that maternal supplementation during pregnancy and/or lactation support the role for LCPUFA in the neurodevelopment of infants [256]. Supplementation with LCPUFA-containing infant formula for more than 6 months increased the likelihood of observing improved cognitive function during infancy [256,257].

The reasons that some studies failed to show a statistically significant association between LCPUFA intake and better neurological performance may have been related to limitations of study design and the use of varying amounts and sources of LCPUFA. For example, in the United States, most of the recent studies that have considered both neurocognitive function and growth have used infant formulas for preterm and term infants that contain DHA and ARA from single cell oils (DHASCO^®^ and ARASCO^®^). Outside the United States, DHA may be obtained from fish oil [258,259] and ARA may be obtained from eggs [260,261]. As mentioned above, the early studies of the 1990s used a variety of experimental formulas with different sources and amounts of LCPUFA (many of these studies are cited by EFSA in their review, [71]). This is noteworthy because only DHA and ARA from single cell oils have been recently shown to enhance neurodevelopment in both preterm [262,263,264] and term infants [257,265]. The source of the oil is important because it significantly affects growth. Clandinin *et al.* [264] have shown that body weight at 118 days and length at 79 and 92 weeks of age in preterm infants fed formula containing ARA and DHA from single-cell oils were greater than in those than those fed a formula containing DHA from tuna oil. Additionally, for both weight and length, there were no differences between breastfed infants and those fed ARA and DHA from single-cell oils [264]. The possibility that ARA and DHA derived from single-cell oils or fish oil have differing effects on growth and neurodevelopment may also be due to the EPA at too high a level in fish oil and its propensity to antagonize ARA [257]. An ARA:DHA ratio greater than 1:1 with up to 0.65% of ARA of total fatty acids is associated with improved cognitive performance [255] and balances the potential competition caused by high levels of DHA.

## 12. The Benefits of ARA for Infant Health

LCPUFA are not only important for growth and neurodevelopment, but recent studies have shown that LCPUFA are also critical for infant health. In an early study, infants fed formula with ARA and DHA developed significantly less stage II and III NEC than those fed a control formula but had similar rates of bronchopulmonary dysplasia, septicemia and retinopathy of prematurity (ROP) [266]. In a recent retrospective cohort study of premature infants (<30 weeks gestation), the relationship between fatty acid profiles during the first postnatal month and infant morbidity due to chronic lung disease, ROP, and late-onset sepsis was analyzed [120]. Results indicated that fatty acid levels of DHA and ARA declined rapidly with a concomitant increase in LA. While the decreased DHA level was associated with increased risk of chronic lung disease, decreased ARA was associated with increased risk of late-onset sepsis. The authors noted that in premature infants, low levels of DHA and ARA contribute to dysregulation of immune and inflammatory responses, predisposing these infants to chronic lung disease and late-onset sepsis [120]. The DHA-derived resolvins decrease neutrophil infiltration and enhance macrophage phagocytosis [267]. DHA also downregulates nuclear factor kB activity in cells either directly or by stimulating the activation of peroxisome proliferator-activated receptors thereby limiting the pro-inflammatory signaling mediated by ARA [267]. Thus, a low level of DHA would predispose these infants to an increased inflammatory response as seen in chronic lung disease. In late-onset sepsis, a decreased ARA level increases the risk of inhibiting the innate immune response through decreased production of eicosanoids, particularly leukotrienes, which enhance chemotaxis of leukocytes, neutrophil activation, and activity of natural killer cells [97,120]. As a consequence, in premature infants, a balance of DHA and ARA levels must be maintained to help reduce the development of morbidity due to prematurity.

Studies have demonstrated that LCPUFA in human milk can modulate immunological responses and affect the T-helper cell (Th) type-1 (Th1)/Th2 balance [268,269]. Th1 cell effectors produce interferon-γ (IFN-γ) and TNF-α which regulate cellular immunity against infection whereas Th2 cells produce interleukin (IL)-4, IL-5, and IL-13 which help mediate immunity against parasitic infections [270,271]. For example, Barakat *et al.* [272] reported that supplementation for 14 days with 10 mg/kg of ARA in *Schistosoma mansoni*-infected schoolchildren induced moderate cure rates (50%) in children with light infection and modest cure rates (21%) in those with high infection. The cure rates associated with ARA were comparable to those produced by 40 mg/kg of praziquantel. The combination of ARA and praziquantel elicited 83% and 78% cure rates in children with light and heavy infections, respectively [272].

The relationship between maternal fatty acid desaturase (FADS) genotype and LCPUFA levels in human milk on infant blood T-cell profiles and cytokine production in 6-month old infants was recently considered [271]. LCPUFA levels in human milk were measured at 4 weeks of age and the FADS genotype was determined in both mothers and infants. Results indicated that ARA levels in human milk were inversely correlated with the production of the cytokines IL-10, IL-17, IL-5 and IL-13 and EPA levels were positively associated with counts of regulatory T-cells and cytotoxic T-cells and decreased T-helper cell counts. The minor FADS alleles were associated with lower ARA and EPA levels in human milk and a higher production of IL-10, Il-17, and IL-5. The major FADS alleles were associated with an increase in the level of ARA in human milk (19%–22%) compared with the minor alleles. There were no association between T-cell distribution and maternal or infant gene variants. Also, there was no relationship between cytokine levels in plasma and levels of LA, EPA, or DHA in human milk.

It has been shown that the FADs gene polymorphism may influence the risk of developing allergies in children [273]. In the study by Muc *et al.* [271] the strongest association between LCPUFA levels and cytokines was observed among those related to the activity of type-2 and type-17 *vs.* those from type-1 responses. The expression of type-2 and type-17 cells have been linked to increased airway inflammation in severe asthma [274]. By reducing type-2 and type-17 activity, LCPUFA including ARA found in human milk may help reduce the risk of childhood asthma and allergies [271].

Notably, Th17 cytokines were initially identified as key factors in the induction of inflammation and tissue destruction associated with a variety of autoimmune response such as multiple sclerosis, arthritis, colitis, celiac disease, and gluten sensitivity [275,276]. However, it is becoming apparent that T17 cells also provide protective immunity against various pathogens at different mucosal sites [270]. Thus, there is a fine balance between protection and pathological manifestation of Th17 responses. As a consequence, a balance of LCPUFA levels similar to that found in human milk is needed to help reduce the risk of developing autoimmune diseases in childhood.

Two studies have compared the frequency of common illnesses in infants fed formula with and without DHA and ARA [277,278]. Both studies used the same LCPUFA-supplemented formula that contained 0.32% DHA and 0.64% ARA of total fatty acids (17 mg of DHA/100 kcal and 34 mg of ARA/100 kcal). In the first study, infants fed the LCPUFA-supplemented formula experienced a lower incidence of bronchiolitis/bronchitis compared with infants fed formula without DHA and ARA [277]. The results from the second study were similar to those of the first. Infants who consumed formula with DHA and ARA had a lower incidence of bronchiolitis/bronchitis, nasal congestion, cough and diarrhea requiring medical attention than infants fed formula without DHA and ARA. The authors indicated that DHA and ARA at present levels in infant formula and follow-on formulas may have a positive effect on moderate to severe common infant illnesses, including diarrhea [277,278].

Two cohorts of children who had previously completed randomized, double-blind trials (one published [72], one unpublished) in which they received a LCPUFA-supplemented formula that contained 0.32%–0.36% DHA and 0.64%–0.72% ARA of total fatty acids or an unsupplemented formula (control) fed during the first year of life were followed up to 3 years of age to determine the incidence of allergies and common respiratory illnesses [279]. The LCPUFA-supplemented group had a significantly lower risk for developing upper respiratory infections, wheezing/asthma, atopic dermatitis, any allergy, and a longer time to first diagnosis than those given an unsupplemented formula.

A subset of children from the Kansas City cohort of the DIAMOND (DHA Intake and Measurement of Neural Development) study [96] were followed to 4 years of age to determine the incidence of childhood allergies [280]. As infants, they were fed either a control unsupplemented formula or one of three formulas with either 0.32%, 0.64% or 0.96% of total fatty acids as DHA with the same amount of ARA (0.64% of total fatty acids). All the different DHA dose and ARA supplemented subjects were pooled into a single supplemented cohort. Results indicated that the incidence of allergic illnesses in the first year of life was lower in the combined LCPUFA group compared with the control. By 4 years of age, LCPUFA supplementation significantly delayed time to first allergic illness and skin allergic illness. LCPUFA supplementation also reduced the risk of any allergic diseases and skin allergic diseases. If the mother had allergies, LCPUFA supplementation reduced the incidence of wheezing/asthma in her offspring. The results of these allergy studies add to the evidence that supplementation of infant formula with both ARA and DHA in the first year of life delays the onset of allergy and may have a protective effect against allergy in early childhood.

Crawford *et al.* [45] reviewed the potential role that ARA and DHA play in protecting against some central nervous system injuries in preterm infants. Deficits of ARA and DHA may contribute to the complications related to prematurity [45]. The mechanism of action responsible for central nervous system injury is reduced vascular or endothelial integrity leading to hemorrhage or ischemia. ARA acts as an endothelial relaxation factor and plays a dominant role in endothelial membrane lipids. The inner cell membrane lipid of the endothelium is especially rich in ARA which provides for membrane properties, signaling and protein kinase C activation [281]. ARA is also a precursor for a range of small molecules that play a key role in cell trafficking, communication and vaso-regulation. As a result, any ARA or DHA deficiency in very preterm infants will be exacerbated after birth during a period of rapid growth. This deficiency may then lead to fragile, leaking vessels and rupture as seen in ROP [45].

The proportion of ARA the placenta delivers to the fetus ranges from 14% to 20% [45]. Infant formula for preterm infants only delivers ~0.4%–0.6% of ARA. For DHA, the placenta delivery to the fetus is ~6% whereas infant formula delivers ~0.3%. That is a 50-fold reduction in ARA and a >10-fold reduction in DHA compared with the infant’s apparent physiological need. When plasma levels of ARA and DHA are followed from birth, they continue to decrease to about one third of that of the fetus. Crawford *et al.* [45] argue that higher intakes of ARA and DHA are needed to correct for deficiency during the first year of life.

To explore the impact that deficiency of ARA and DHA may have on immune cell function Moodley *et al.* [282] examined the fatty acid profile and main phosphoglyceride content of cord blood mononuclear cell (CBMC) membranes in healthy preterm infants (30 to 35 weeks) and term infants (37 to 40+ weeks). Results indicated that ARA was the dominant LCPUFA present in both PC and PE membrane fractions of CBMCs in both preterm and term infants. The proportions of ARA, DHA and other LCPUFA were significantly lower in PE and PC of preterm infants compared with those in term infants. The dominance of ARA was consistent with the process of biomagnification that preferentially selects ARA rather than other LCPUFA for transfer to the fetus. Preterm infants also had significantly lower absolute numbers of CD4+ leukocytes and CD4+ and CD8+ naïve T-cells. At birth, there is a period of transition from a sterile environment to one of higher infectious risk. The elevated levels of ARA in CBMCs concomitant with lower levels of other LCPUFA suggest that the acquisition of ARA is needed in preparation for a responsive immune system after birth. These findings indicate that in preterm infants a deficiency in the supply of ARA exists which may compromise their immune system [282].

Infants with mildly abnormal physical movements at 12 weeks of age are reported to have lower ARA content in erythrocyte membranes [283]. This abnormality occurred with maternal supplementation of DHA alone but was not seen when DHA was combined with ARA during pregnancy and lactation [283]. Mildly abnormal movements have been also observed during infancy and linked to increased prevalence of minor neurologic dysfunction and attention deficits at school age [284]. These findings imply that during early brain development of neonates, a supply of ARA is critical.

The effects that feeding preterm infants human milk (HM), infant formula without DHA and ARA (F) or formula with DHA (0.35%) and ARA (0.49%) have on isolated peripheral blood lymphocytes and lipid composition was evaluated by Field *et al.* [285]. Adding DHA and ARA to a preterm infant formula resulted in lymphocyte and cytokine production, phospholipid composition, and antigen maturity similar to those observed in infants fed human milk. These findings suggest that the addition of both DHA and ARA may improve the ability of the infant to respond to immune challenges in a manner similar to breastfed infants [285].

Several epidemiological studies have shown that individuals with learning disorders including attention deficit hyperactivity disorder (ADHD), dyslexia, and autism have signs of EFA deficiency or have lower than normal blood levels of DHA and ARA [286,287,288,289]. A meta-analysis of pooled data from RBC and plasma/serum samples indicated that ARA and DHA concentrations were significantly lower than normal in individuals with learning/developmental disorders [290]. In absolute amounts, the level of ARA was as severely depressed as DHA within RBC (both ~0.58 mg/100 mg of fatty acid below normal) but much lower than DHA within plasma/serum (−0.71 *vs.* −0.34). The reason for lower than normal blood levels of ARA in children with learning disorders is unknown but could be related to a low dietary intake of ARA relative to metabolic requirements or that ARA is not synthesized efficiently from precursor fatty acids, or not delivered or properly incorporated into the brain [290].

The effects of subnormal ARA on brain function seems to be independent of those associated with *n*-3 deficiency. In Japan where intakes of *n*-3 fatty acids are relatively high, the incidence of dyslexia in children is similar to that observed in Westernized countries (~6%) [291]. Therefore, although the Japanese population consumes sufficient amounts of *n*-3 fatty acids, there still may be insufficient intake of ARA to meet the needs for normal brain function during childhood [290]. The important role of ARA in normal growth and development requires as much research emphasis as DHA has received. ARA should be the focus of preclinical and clinical research for a detailed assessment of dietary requirements.

## 13. The Regulatory Requirements for ARA and DHA in Infant Formulas

A joint International Expert Consultation of the Food and Agricultural Organization of the United Nations (FAO) and the WHO was assembled in 1976 to review the literature on “The Role of Dietary Fats and Oils in Human Nutrition” [292]. The section dealing with infant growth and development indicated that the ideal recommendation for infant formulas would be to match the essential fatty acids found in human milk with respect to LCPUFA content [292]. The FAO further stated that LCPUFA were particularly important during fetal and infant growth when there is a high demand for the synthesis of cell structured lipid [292]. FAO issued a follow-up report in 1994 which provided additional supportive recommendations for adding both ARA and DHA to infant formulas. The FAO stated that “the *n*-6 and *n*-3 fatty acids have critical roles in the membrane structure and as precursors of eicosanoids, which are potent and highly reactive compounds. Various eicosanoids have widely divergent, and often opposing effects on, for example, smooth muscle cells, platelet aggregation, vascular parameters (permeability, contractility), and on the inflammatory processes and the immune system. Since they compete for the same enzymes and have different biological roles, the balance between the *n*-6 and the *n*-3 fatty acids in the diet can be of considerable importance” [293]. In a follow-up report in 2008–2010, the FAO/WHO Expert Consultancy on Fats and Fatty Acids further concluded that “There can be little doubt about the essentiality of DHA and ARA for the brain” [294].

A global standard for infant formula was established by the Codex Alimentarius Commission in 1981, and revised over the years [295]. The latest revision was issued in 2007 and the latest amendment was added in 2015 [296]. The standard includes details on essential composition of nutrients and a list of food additives that are allowed to be added. Quality control measures such as labeling, packaging, contaminants and hygiene are also specified. In the United States, standards for infant formula are the responsibility of the U.S. Food and Drug Administration (FDA). The U.S. Code of Federal Regulations Title 21, Part 106 specifies infant formula quality control procedures and Part 107 lists the nutrient requirements and other rules concerning labeling for infant formulas. Not surprisingly, the quality and safety standards for infant formulas are extremely high, exceeding most requirements for other food products [297].

In the European Union (EU), the legislation on infant formula and follow-on formulas was adopted in 2006 [298] and at the time of this writing is being revised. Before revising the legislation the European Commission requested the European Food Safety Authority (EFSA) Panel on Dietetic Products, Nutrition and Allergies to provide their scientific advice on the essential nutrient composition of infant and follow-on formulas [70,71]. In the first report of 2013, dedicated to nutrient requirements and dietary intakes of infants and young children in the EU, the EFSA Panel reviewed a variety of nutrients, including the levels of DHA and ARA. In the 2013 EFSA report, adequate intakes were defined as 100 mg/day of DHA and 140 mg/day of ARA from birth to six months of age. From 6 to 24 months of age, 100 mg/day of DHA were considered adequate. These recommendations were also supported by a global expert panel, based on a systematic review of the available scientific literature [299]. However, in the subsequent report of 2014, dedicated to essential composition of infant and follow-on formulae, the EFSA Panel advised that infant and follow-on formulas should contain relatively higher amounts of DHA (20–50 mg/100 kcal). Mandatory addition of ARA was not supported in this EFSA report. Still, the Panel noted that feeding an infant formula containing DHA alone resulted in lower concentration of ARA in erythrocytes compared with a control formula without DHA.

At an assumed mean fat content of 5.2 g 100 kcal in a typical infant formula, this means that the recommendation of higher levels of DHA would result in a DHA content of 0.38% to 0.96% of fatty acids, higher than the 0.2% to 0.3% of DHA currently found in most infant formulas available in the marketplace [2]. Notably, PUFA-supplemented commercially available infant formulas contain preformed ARA at levels equal to or higher than the DHA content. The ESFA Panel’s advice of providing up to 1% DHA and no ARA is a unique approach and directly opposite to a consensus reached by international expert groups who have recommended that infant formulas for term infants should contain ARA at levels that range from 0.4% to 0.7% fatty acids (at a 1:1–2:1 ratio to DHA) based on the median worldwide range of ARA and DHA concentrations in breast milk [6,7,8,9].

## 14. Discussion

ARA is the principle LCPUFA in the inner cell membrane lipid of muscle, heart, vascular endothelium, adrenals, kidneys, liver, the placenta, and in almost all other organs [300]. ARA is essential for cell integrity. The cell membrane separates the interior structures of cells from the outside environment. It also controls the movement of substances in and out of the cell [300]. These membranes contain signalers, receptors, ion channels, antioxidant defense enzymes, and rafts. Changing any aspect of the composition of the cell membrane may alter its function [300]).

ARA has very different biological functions than DHA [300]. While DHA controls signaling membranes in the photoreceptor, brain and nervous system, ARA is indispensable in the vasculature and in specific aspects of immunity. ARA is important for brain growth during gestation and early infancy where it plays a critical role in cell division and signaling [11]. A potentially important aspect of ARA metabolism is its function as a precursor for leukotrienes, prostaglandins, and thromboxanes, collectively known as eicosanoids. Eicosanoids have numerous critical and specific functions occurring in almost every tissue of the body. Eicosanoids function to modulate the release of somatostatin, the principal hormone that stimulates cell proliferation and growth. Eicosanoids also have important roles in immunity and inflammation.

This review focused on the essentiality of ARA for infant growth and development. Animal studies demonstrated the importance of ARA for growth and maturation of neurons and myelin and the resolution of inflammation in models of NEC, influenza and EFA deficiency. These studies also provided compelling evidence that both preformed DHA and ARA are required for optimal cognitive and neurological development. The ratio of ARA and DHA added as supplements really matters. Brain tissue analysis of neonatal baboons fed formula with a high level of 0.96% DHA significantly reduced ARA levels in two regions of the brain indicating the importance of a proper balance of DHA and ARA [24].

For over 10 years, both DHA and ARA have been added to infant formulas worldwide in an attempt to match the nutrient supply and functional benefits achieved with human milk. The combination of ARA and DHA in infant formulas has been shown to be safe in many millions of infants globally. The DHA concentration in human milk is lower and more variable than for ARA and the level of ARA in human milk is more stable [5]. The relatively stability of the ARA level in human milk is biologically important because it provides preformed ARA consistently at a time when brain growth and development is most critical [300]. Although DHA is more variable than ARA it is always present in human milk and the balance between ARA and DHA can be as much as 2 to 1.

The biosynthetic capability for providing ARA and DHA for brain growth is low and preformed ARA and DHA are preferentially incorporated into the brain during gestation and early infancy [2,46]. Infant formulas devoid of ARA results in a dramatic decrease of up to 40% of ARA in plasma shortly after birth [223,230,238,239,300,301], especially in preterm infants who do not receive the third trimester’s maternal supply of ARA and DHA. The finding that there is a decrease in ARA shortly after birth shows that biosynthetic capability is insufficient to meet the infant’s demand [300]. The process of biomagnification and its resulting fatty acid profile further highlights the importance of ARA in infant growth [45]. In several clinical studies, the provision of high amounts of DHA/EPA without a concomitant supply of ARA has been associated with adverse effects on growth in premature infants [240,241,242,243].

EFSA [71] recently concluded that “there is no necessity to add ARA to infant formula even in the presence of DHA”. This recommendation needs further explanation. One of the possible reasons for this recommendation is that it is generally believed that LA is converted into ARA in sufficient quantities, even though EFSA noted that feeding an infant formula containing DHA alone resulted in lower concentration of ARA in erythrocytes compared with a control formula without DHA. From the limited dietary intake data presented here for non-breastfed infants and young children (Table 1) living in developing and developed countries there is evidence that intakes of ARA from dietary sources are very low, much lower than the average amount of ARA available in human milk or infant formulas containing ARA and DHA. The composition of infant formulas and follow-on formulas should therefore not only be based on human milk composition but also on food/nutrient intake data to address the assumption that complementary foods fill nutrient gaps.

The EFSA Panel’s advice of providing higher amounts of DHA (20–50 mg/100 kcal, 0.38% to 0.96% of fatty acids) without a concomitant supply of ARA is also questionable. As discussed here, DHA suppresses ARA concentration in membranes and its function. As a result, an infant formula with DHA and no ARA may result in a potential higher risk of morbidity due to the suppression of favorable eicosanoids that play a key role in cell trafficking, communication and vaso-regulation [45].

EFSA did not take into account the original FAO/WHO publications as well as the earlier 2008–2010 publication which strongly concluded “There can be little doubt about the essentiality of DHA and ARA for the brain” [294]. When infants are exclusively breastfed during the first 6 months of life, “there is evidence of a requirement for preformed ARA and DHA after 6 months of life” [294]. According to the 2010 FAO/WHO statement, DHA and ARA should be included in infant formula with DHA (from 0.2% to 0.5% of total fatty acids) and added ARA should be at least equal to the amount of DHA [294].

The clinical trials considered by EFSA were not designed to consider the specific physiological outcomes related to ARA. Most studies included both ARA and DHA. There were no clinical trials that evaluated the effects of ARA in the absence of DHA. The benefits of ARA + DHA supplementation cannot be ascribed to DHA alone but logically must be ascribed to the variables used in most of these studies, the combination of the two. The combination of ARA and DHA has shown benefits for cognitive development, visual function, and blood pressure well beyond the period of supplementation and into early childhood [249,302].

EFSA’s lack of support of ARA in DHA-containing formulas relied almost exclusively on the meta-analysis of infant growth conducted by Makrides *et al.* [303]. However, the meta-analysis of Makrides *et al.* [303] was not comprehensive. The majority of subjects (*n* = 1050) included in the meta-analysis participated in 8 clinical trials using formula containing both DHA and ARA. Only 341 subjects in the 8 trials were provided infant formula with DHA/EPA without ARA. At 12 months of age, only 99 subjects were supplemented with DHA in the absence of ARA. Additionally, the analysis excluded studies that included preterm infants who are most vulnerable to growth faltering due to nutrition, and excluded studies in which DHA plus ARA supplementation was less than 3 months during which growth velocity is particularly sensitive to nutritional inadequacy. The reason for excluding these studies was not reported.

In the meta-analysis, Makrides *et al.* [303] noted that the results were inconclusive, *i.e.*, LCPUFA supplementation had no detrimental effect on growth. The importance of ARA as a structural and metabolically active lipid, was not addressed. Better visual and mental performance was attributed to the contribution of DHA. However, in one of the studies considered, DHA plus ARA improved mental function [265] compared with an unsupplemented control. In fact, in one study, DHA alone did not perform better than did the unsupplemented controls [72].

Clinical evidence to support the safe removal of ARA from infant formula and follow-on formula containing DHA is lacking. The human and nonhuman primate studies described herein question the EFSA recommendation to provide infant formula from birth with up to 1% of DHA without a proportional amount of ARA [2]. Any major change in infant formula composition should be subjected to a full preclinical and clinical evaluation of safety and nutritional adequacy before its introduction into the marketplace [2]. Without such an assessment, and in light of the universal presence of ARA in human milk and the numerous essential ARA functions for cell structure and function, the most judicious approach is to include ARA in DHA-containing infant formulas to promote optimal infant growth and development.

## Figures and Tables

**Table 1:** ARA intakes in developed and developing countries during the first 2 years of life.

Country	Age	Method	Mean ARA Intake (mg/Day) (mg/kg/Day) ^1^
Australia [91]	2–3 years	1-day weighed food record	16 (1.3)
		1-day weighed food record	22 (1.8)
Belgium [90]	2–5 years	3 days food record	17 (1.4)
	4–6.5 years	3 days food record	18 (1.0)
Canada [92,93]	1.5–2 years	1 day food frequency	133 (11.0)
	2.1–3 years	I day food frequency	260 (22.0)
	3.1–5 years	1 day food frequency	226 (15.0)
	4–7 years	3-days food records	57 (2.9)
China [82]	1–3 years	3 days 24 h recall	55 (4.6)
	4–5 years	3 days 24 h recall	50 (2.5)
Gambia [82]	0–6 months	1 day weighed food monthly	90 (15.0)
	7–12 months	1 day weighed food monthly	70 (7.8)
	13–17 months	1 day weighed food monthly	60 (6.7)
	24 months	1 day weighed food monthly	10 (0.8)
Germany [87,88]	6 months	3 days weighed food record	72 (12.0)
	9 months	3 days weighed food record	24 (2.7)
Italy [89]	1 month	Human milk composition	95.6 (29.0)
	2 months	Human milk composition	109.6 (33.0)
	3 months	Human milk composition	101.1 (16.9)
	6 months	Human milk composition	58.7 (9.8)
U.S. 2003–2008 [94]	1–4 years	1 day weighed food record	60 (5.0)
U.S. 2015 [95]	2–5 years	1 day weighed food record	80 (6.7)

Notes: ^1^ Estimated mean intake for ARA (9 mg/kg/day) for ages 0 month to 3 years was calculated using median weight-for-age percentiles for boys, birth to 36 months, and from median body mass index for ages 4 through 19 years; from the Centers for Disease Control and Prevention-Growth Charts (CDC, [78]).

**Table 2:** Pro- and anti-inflammatory effects of prostaglandin E_2_ (PGE_2_) and leukotriene B_4_ (LTB_4_) ^1^.

Eicosanoid	Effects
PGE_2_	**Proinflammatory**
	Induces fever
	Increases vascular permeability
	Increases vasodilatation
	Causes pain
	Enhances pain caused by other agents
	**Anti-inflammatory**
	Inhibits production of TNF and IL-1
	Inhibits 5-LOX (decreases 4-series LT production
	Induces 15-LOX (increases lipoxin production)
LTB_4_	**Proinflammatory**
	Increases vascular permeability
	Enhances local blood flow
	Chemotactic agent for leukocytes
	Induces release of lysomal enzymes
	Induces release of oxygen species by granulocytes
	Increases production of TNF, IL-8, and IL-6

Notes: ^1^ IL, interleukin; LOX, lipoxygenase; TNF, tumor necrosis factor. From Calder [98].

**Table 3:** Roles of ARA in bone formation, metabolism, and mineral balance.

Metabolic Effector	Physiological Roles of ARA
ARA [149]	Maintain normal balance between bone mineral accrual and bone resorption during infant development
ARA, growth hormones [150,151]	Increase insulin-like growth factor gene expression and induction of osteoblast-dependent bone formation
Vitamin D_3_ [128,133]	Mediate vitamin D_3_ coordination of chondrocyte proliferation in the epiphyseal growth plates of long bones. Parathyroid hormone secretion
Calcium and phosphorous [152]	Regulation of parathyroid hormone secretion in response to blood mineral concentrations
Parathyroid hormone [149,150]	ARA mediated/activated pathway involved in the activation and secretion of vitamin D_3_ by kidneys
Physical activity [153]	ARA mediates bone adaptation to changes in physical stress through mechanisms which mediate resorption and remodeling
